# Gallery-Distance Profiles for Open-Set Person Re-Identification with Frozen Encoders in Camera Networks

**DOI:** 10.3390/s26175479

**Published:** 2026-08-29

**Authors:** Jin Wang, Jiannong Cao, Changlin Song

**Affiliations:** 1 College of Internet of Things Engineering, Wuxi University, Wuxi 214105, China; 2 Department of Computing, The Hong Kong Polytechnic University, Hong Kong, China; jiannong.cao@polyu.edu.hk (J.C.); charlemagnescl@outlook.com (C.S.)

**Keywords:** camera networks, person re-identification, open-set recognition, unknown-person detection, adaptive decision, model selection, distance distribution, episodic learning

## Abstract

Open-set person re-identification (ReID) must determine whether a query identity is represented in the current gallery before returning a cross-camera match. We investigate whether the complete query-to-gallery distance distribution provides more reliable presence evidence than its nearest value when the encoder is frozen and target unknown identities are unavailable. The proposed Source-Episodic Distance-Statistics Evaluator (SDSE) projects each distance vector onto nine interpretable local, global, and candidate-conditioned statistics. Auxiliary identity episodes reproduce the gallery-presence hypotheses and fit a low-capacity evaluator; leave-one-out scores from the enrolled target gallery calibrate deployment without changing the encoder or accepted-query ranking. Across Market-1501, DukeMTMC-reID, and CUHK03, SDSE gives the strongest macro unknown-detection result among seven frozen-embedding scorers. A 20-split paired analysis confirms a 0.0095 gain in area under the receiver operating characteristic curve (AUROC) over nearest distance, with the clearest benefits under cross-camera-domain transfer. Controlled gallery perturbations preserve the relative advantage but show that sparse identity evidence limits absolute performance. Grouped-profile experiments further identify the combination of local and global distribution evidence as the main source of the gain. An auxiliary operating-point gate and a six-candidate encoder-selection experiment illustrate how the common profile interface can support deployment decisions, while their measured scope is kept separate from the central scoring contribution. The results establish SDSE as a lightweight gallery-relative decision layer for fixed-gallery open-set ReID rather than a substitute for representation learning.

## 1. Introduction

Person re-identification (ReID) associates a person observed by one camera with gallery images collected by other cameras. Standard benchmarks formulate this task as closed-set retrieval: every query is assumed to have a valid gallery match. Camera networks violate that assumption whenever non-enrolled people enter the scene. A nearest-neighbor system must nevertheless return an identity, so a visually plausible impostor can become a false match. The risk is amplified when deployment cameras differ from the representation-training environment in viewpoint, illumination, background, resolution, or detector quality [1,2].

This study asks a specific scientific question: when the ReID representation is fixed, can the full query-to-gallery distance distribution reveal gallery membership more reliably than the nearest distance alone? The target problem is gallery-relative open-set ReID. Each query must either be linked to a valid enrolled cross-camera identity or rejected because its identity is absent from the current gallery. The question is deliberately downstream of representation learning: the encoder and gallery ranking remain unchanged, and target unknown identities are unavailable for learning or calibration.

Nearest-distance rejection is a natural starting point because nearest-neighbor evidence is effective for novelty detection in deep feature spaces [3]. Its limitation is statistical: one extreme observation stands in for hundreds or thousands of gallery comparisons. A difficult enrolled query can have a relatively large nearest distance yet retain a coherent identity-specific neighborhood. Conversely, a non-enrolled query can acquire one accidental close match while remaining inconsistent with both that candidate identity and the wider gallery. These cases suggest treating identity presence as a distributional hypothesis test rather than a one-distance threshold test.

The Source-Episodic Distance-Statistics Evaluator (SDSE) answers this question through a fixed nine-dimensional profile. Lower-tail order statistics describe proximity and ambiguity; global location, spread, and upper-tail summaries locate the query within the complete gallery distribution; and a candidate-conditioned dispersion contrast measures the coherence of the nearest identity. A class-balanced logistic evaluator combines these signals without changing the encoder. Grouped-profile, nested-profile, and leave-one-feature-out experiments test which parts of the design carry the evidence, rather than assuming that nine is an optimal dimension.

The protocol separates evaluator fitting, threshold calibration, and target evaluation. Auxiliary episodes provide known and pseudo-unknown distance profiles, with the two identity sets disjoint within each episode. A regularized logistic evaluator is fitted to these profiles. Leave-one-out scores from the enrolled target gallery then calibrate the rejection threshold, while official target queries are reserved for evaluation. A separate gallery internal resampling procedure is used to compare frozen encoders. Neither procedure uses target unknown labels.

SDSE produces a continuous unknown-risk score. A separate Unknown-Candidate Gate (UCG) examines the binary operating point through gallery-distance energy and diagonal Mahalanobis deviation, while the fixed-dimensional profile also supplies a common interface for a gallery-only encoder-selection experiment. These two deployment analyses are evaluated independently of the central score: UCG does not alter threshold-free ranking, and candidate selection occurs before official target queries are observed.

Our 2023 arXiv preprint introduced the nine-statistic representation and the candidate-positive/candidate-negative coefficient-of-variation (CV) contrast [4]. Table 1 distinguishes that representation from the present gallery-presence formulation, source-episodic evaluator, target-known calibration, official Rank-1, mean average precision (mAP), and open-set classification rate (OSCR) protocol, and deployment analyses.

The contributions form a single claim–mechanism–evidence chain:

- We formulate frozen-encoder, gallery-relative open-set ReID as a distributional presence test and derive three observable evidence groups from the known/unknown mixture structure of query-to-gallery distances.
- We realize this principle as SDSE: an interpretable fixed-dimensional profile, a low-capacity evaluator fitted from identity-disjoint auxiliary episodes, and target-known-only calibration. The ReID representation is never adapted, so accepted-query ranking is preserved.
- We connect each design claim to paired evidence: seven post hoc scorers, 20 identity splits with bootstrap and non-parametric inference, grouped and nested profile analyses, and controlled perturbations of gallery size, samples per identity, imbalance, and query prevalence.
- We also use the profile for operating-point control and pre-query encoder selection. These analyses identify both the useful mechanism and its boundary: energy explains the observed UCG behavior, and model-selection evidence is confined to the tested six-candidate bank.

**Figure 1:**
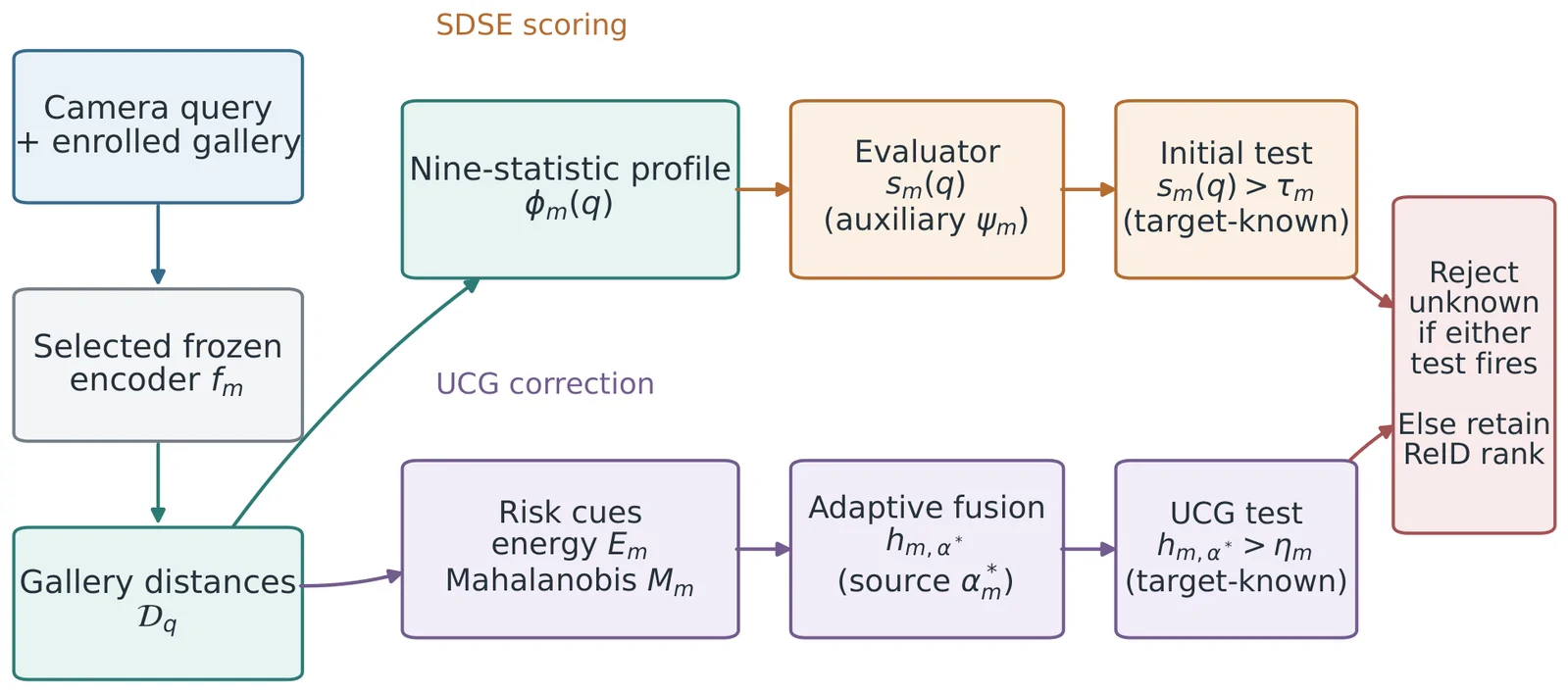
Gallery-conditioned SDSE and adaptive UCG. A selected frozen encoder supplies one query-to-gallery distance vector. Its nine-statistic profile produces the continuous SDSE score, while gallery-distance energy and diagonal Mahalanobis deviation form the auxiliary risk gate. Auxiliary data train $\psi_{m}$ and select $\alpha_{m}^{*}$; the target known gallery calibrates $\tau_{m}$ and $\eta_{m}$. Final rejection is the union of the two tests, and accepted-query ranking remains unchanged.

**Figure 2:**
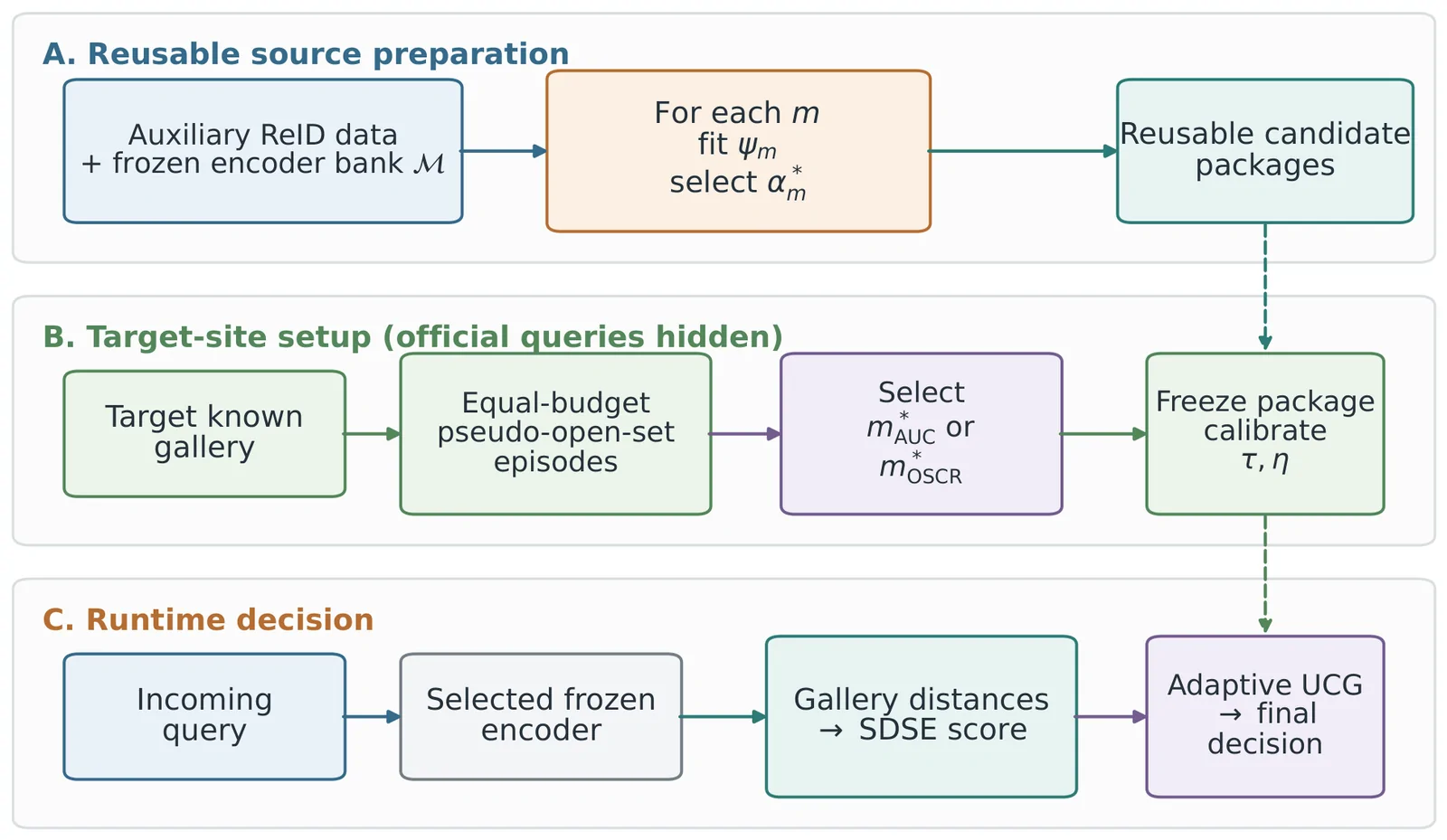
End-to-end deployment pipeline. Auxiliary data preparation $\left(\psi_{m},\alpha_{m}^{*}\right)$ for each frozen candidate; equal-budget target-gallery episodes select an encoder without official target queries; the selected encoder and evaluator are then calibrated from the known gallery. At runtime, SDSE ranks unknown risk, and adaptive UCG can reject only high-risk accepted queries.

Figure 1 gives the component-level view of the gallery-conditioned score and auxiliary gate, while Figure 2 places these components in the complete selection, calibration, and deployment pipeline.

**Figure 3:**
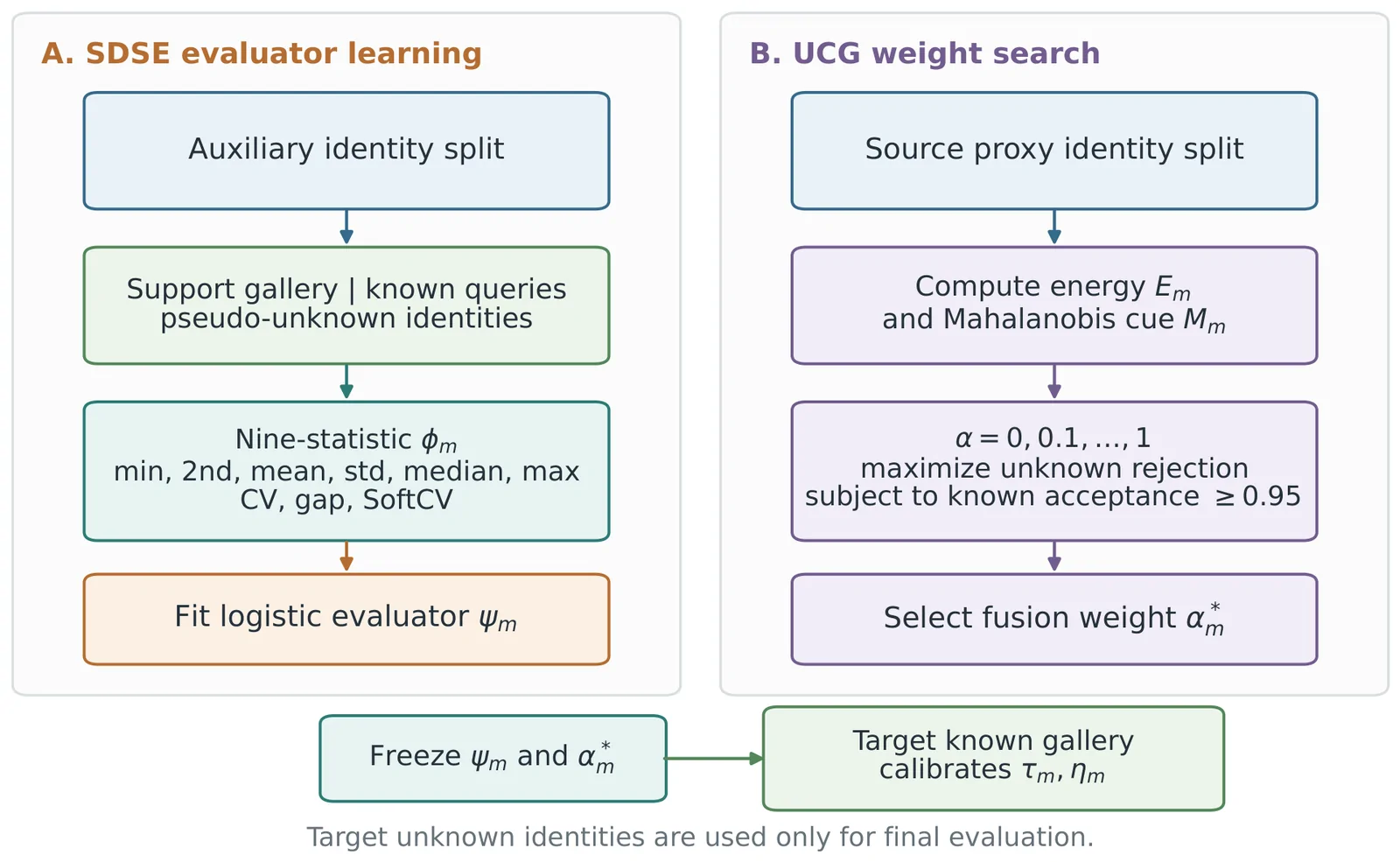
Distinct uses of auxiliary supervision. Source episodes train the SDSE evaluator from nine-statistic profiles, whereas source proxy splits search over the UCG energy weight under a known-acceptance constraint. Both $\psi_{m}$ and $\alpha_{m}^{*}$ are frozen before the target known gallery calibrates $\tau_{m}$ and $\eta_{m}$; target unknown labels are evaluation-only.

Figure 3 separates the three uses of data: evaluator learning, gate-weight selection, and target-known calibration. Together, they make explicit which quantities are learned, which are calibrated, and which remain frozen.

## 2. Related Work

### 2.1. Closed-Set and Open-World Person Re-Identification

Closed-set ReID learns an embedding that brings cross-camera images of the same person together while separating different identities. Cross-domain methods improve transfer through image translation, pseudo-labeling, clustering, feature alignment, or domain-general representation learning [1,2,5,6]. OSNet, used in our main experiments, aggregates multiple receptive scales in an efficient feature extractor [7]. These approaches improve representation and ranking, but they normally retain the assumption that every query has a gallery match. A transferable encoder can, therefore, reduce retrieval errors without defining when no enrolled identity is valid.

Liao et al. formalized open-set person ReID as two coupled subproblems: detecting whether the probe identity is present in the gallery and, if accepted, identifying the corresponding person [8]. Their protocol trained general metric learners on independent identities and showed that closed-set retrieval accuracy did not translate into strong low-false-accept open-set performance. This detection–identification coupling is a central distinction between open-set ReID and sample-wise novelty detection.

Subsequent work has mainly strengthened either the representation or the gallery model. In watch-list ReID, Adversarial PersonNet jointly trains a generator, feature extractor, and target discriminator against target-like impostors [9]. DistributionNet represents each image by a feature distribution and learns uncertainty-aware embeddings; its open-world evaluation reports true-target rate at controlled false-target rates [10]. Both approaches alter the learned representation for open-world verification. A different line treats the gallery itself as dynamic: Casao et al. cluster incoming unknown samples and incrementally create or update identity models [11], while recent dynamic benchmarks vary identities, cameras, and time [12].

Existing open-world ReID research, therefore, spans three settings: detection and identification against a static watch list, representation learning that is robust to impostors or uncertainty, and online discovery with gallery expansion. We study a complementary deployment setting in which the encoder and enrolled gallery are fixed, target unknown labels are unavailable, and absent identities must be rejected without changing the ranking of accepted queries. We also consider the preceding deployment decision of choosing a frozen encoder. Table 2 summarizes the differences.

Domain shift and identity novelty are related but distinct. A known query may be difficult because its camera conditions differ from those seen during representation training; an unknown query may resemble an enrolled person even without a domain change. SDSE does not predict domain membership. It estimates whether the query identity is absent from the current gallery, and its evaluation retains the official cross-camera correctness requirement for known identities.

### 2.2. Open-Set Recognition and Adjacent Novelty Detection

Open-set recognition assigns known classes while reserving a rejection outcome for unseen classes. OpenMax fits class-specific extreme-value models to a trained classifier’s activation vectors, whereas PROSER changes classifier training by reserving placeholders for prospective unknown classes [13,14]. These are important representation-and-classifier methods, but neither is a drop-in scorer for a non-parametric ReID gallery: OpenMax requires a fixed activation vector and class-wise calibration, and PROSER requires retraining the classifier and feature space. Treating every enrolled identity as a newly trained parametric class would change the frozen-encoder condition studied here and confound representation changes with post hoc scoring.

The directly comparable family is novelty detection on a fixed reference embedding set. Mahalanobis scoring estimates global deviation from enrolled features, deep nearest-neighbor detection uses local reference distances, and local outlier factor compares local density [3,15,16]. Energy provides a smooth aggregate of distances [17]. The experiments compare nearest distance, mean 5-nearest-neighbor distance, local outlier factor, shrinkage Mahalanobis distance, energy, and unit-normalized cosine distance on identical frozen embeddings and identity splits. This design isolates the unknown-scoring rule while leaving gallery retrieval unchanged.

Unlike fixed-class classification, ReID assigns identities through a replaceable gallery. One identity may have several cross-camera samples, and retrieval rather than a parametric class head determines the predicted identity. A query is, therefore, “known” only relative to the current gallery. The nearest distance retains one extreme observation; SDSE tests whether additional order, distributional, and identity-conditioned evidence improves that rule.

Energy and Mahalanobis scores summarize different forms of incompatibility. UCG searches their weight on auxiliary pseudo-open-set splits and calibrates the threshold from target known-gallery samples. The search is informative only when it can suppress an unhelpful cue; the observed selections favor energy, locating the operating-point effect in that component rather than in general two-cue complementarity.

Three related terms describe different system functions. Unknown-person detection asks whether a query identity is absent from the current gallery. Open-set person ReID combines that decision with the correct identification of accepted enrolled queries under a fixed gallery. Open-world person ReID is broader and may include discovery, temporal association, continual adaptation, and enrollment of previously unknown people. SDSE provides the first function as a score and is evaluated end-to-end on the second; it does not implement the dynamic functions of a complete open-world system.

Evaluation must, therefore, distinguish between score separation, retrieval, and a selected operating point. Table 3 summarizes the reported quantities. AUROC and area under the precision–recall curve (AUPR) measure threshold-free unknown-person detection; the false-positive rate at 95% unknown recall (FPR@95) evaluates one point on the receiver operating characteristic curve. Rank-1 and mAP measure known-person retrieval. OSCR couples detection and retrieval: a known query contributes only if it is both accepted and correctly identified. Known acceptance and unknown rejection describe one calibrated threshold rather than score ranking.

### 2.3. Episodic Learning and Unsupervised Model Selection

Episodic learning varies support and query label sets so that training resembles the intended test-time task [18,19]. PEELER uses novel-class episodes to adapt a few-shot recognizer for open-set decisions [20]. SDSE uses episodic learning in a more restricted way: auxiliary identities generate known and pseudo-unknown distance-profile tasks, but the ReID representation is fixed. Only a low-capacity evaluator is learned over the nine statistics available from any new gallery.

Selecting a model without target labels is difficult because its deployment performance cannot be measured directly. MetaOD transfers performance information from historical outlier datasets and uses dataset meta-features to choose an unsupervised detector [21]. AutoMS instead selects a novelty detector from clean reference data while controlling false discovery through jackknife prediction [22]. Both methods address generic detector or hyperparameter selection; they do not include cross-camera identification correctness or a replaceable identity gallery. Our exploratory selector uses the enrolled ReID gallery itself: cross-camera identities are divided into support samples, pseudo-known queries, and withheld pseudo-unknown queries, producing an internal ranking by the same AUROC or OSCR criterion used at deployment. Real target unknowns and official benchmark queries are not used. Equal identity and episode budgets provide a balanced comparison, and all candidates remain frozen. Its validation is limited to the six candidates described in Section 4.6; broader architectural and source-domain generality remains untested.

Table 4 locates SDSE relative to these neighboring settings. Its role lies at the decision and deployment-selection stages, downstream of representation learning.

## 3. Method

### 3.1. Problem Formulation

Let a frozen encoder, $f_{m}$, from candidate model *m* map an image, *x*, to its exported embedding, $z=f_{m}\left(x\right)\in R^{d_{m}}$. The main Euclidean-distance experiments use these exported vectors without an additional unit-norm transform. When cosine distance is evaluated, we instead use $\bar{z}=z/\left({∥z∥}_{2}+\epsilon \right)$. The target gallery is

(1) $$G^{k}={\left\{\left(g_{i},y_{i},c_{i}\right)\right\}}_{i=1}^{N},$$

where $y_{i}$ is an enrolled identity, and $c_{i}$ is a camera label. Evaluation queries comprise known queries $Q^{k}$, whose identities occur in $G^{k}$, and unknown queries $Q^{u}$, whose identities do not. Known and unknown are, therefore, defined relative to the deployed gallery, rather than as fixed semantic classes: changing $G^{k}$ can change the status of the same identity. For query *q*,

(2) $$d_{i}^{\left(m\right)}={∥f_{m}\left(q\right)-f_{m}\left(g_{i}\right)∥}_{2},\;D_{q}^{\left(m\right)}={\left\{d_{i}^{\left(m\right)}\right\}}_{i=1}^{N}.$$

For a fixed encoder, the query-level problem is to rank valid cross-camera gallery samples for accepted known queries and reject unknown queries. When several pretrained encoders are available, a separate model-level problem is to select one before $Q^{k}$ or $Q^{u}$ is observed, using only auxiliary identities and the enrolled target gallery. This formulation separates open-set inference from deployment-time encoder selection while giving both stages the same gallery-distance interface. For unit-normalized vectors, squared Euclidean distance and cosine distance satisfy ${∥{\bar{z}}_{q}-{\bar{z}}_{i}∥}_{2}^{2}=2\left(1-{\bar{z}}_{q}^{⊤}{\bar{z}}_{i}\right)$ and, therefore, have identical rankings and threshold-free metrics. Nearest-Euclidean distance and SDSE operate on the exported vectors, whereas the cosine baseline normalizes them internally; the two baselines, therefore, test different distance geometries rather than duplicate one another.

The distance vector defines the empirical cumulative distribution function

(3) $${\hat{F}}_{q}^{\left(m\right)}\left(t\right)=\frac{1}{N}\sum_{i=1}^{N}I\left[d_{i}^{\left(m\right)}\leq t\right].$$

Nearest distance retains only the first order statistic. SDSE uses finite projections that describe the lower tail, location, spread, upper tail, and candidate-conditioned structure.

### 3.2. Statistical Rationale: Gallery Presence as a Distributional Test

The open-set decision can be written as a gallery-presence test,

(4) $$H_{0}:y_{q}\in Y_{G},\;H_{1}:y_{q}\notin Y_{G},$$

where $Y_{G}$ is the set of enrolled identities. Let $F_{m,q}^{\mathrm{same}}$ and $F_{m,q}^{\mathrm{diff}}$ denote the query-conditioned distance distributions for same-identity and different-identity gallery samples. If a known query of identity *y* has $n_{y}$ gallery samples, its empirical distance distribution can be viewed approximately as

(5) $${\hat{F}}_{m,q}^{k}\left(t\right)\approx \pi_{y}F_{m,q}^{\mathrm{same}}\left(t\right)+\left(1-\pi_{y}\right)F_{m,q}^{\mathrm{diff}}\left(t\right),\;\pi_{y}=\frac{n_{y}}{N},$$

whereas an unknown query has no same-identity component,

(6) $${\hat{F}}_{m,q}^{u}\left(t\right)\approx F_{m,q}^{\mathrm{diff}}\left(t\right).$$

This is a task-level statistical rationale rather than an independent-and-identically-distributed generative assumption: all distances share the query, and camera, pose, and gallery composition create dependence. The important structural difference is the possible presence of a coherent same-identity component under $H_{0}$ and its absence under $H_{1}$.

The nearest distance is a noisy extreme of this empirical distribution. It can be dominated by one accidental impostor match and tends to change with gallery size even when the underlying representation is fixed. SDSE, therefore, combines three evidence groups, summarized in Table 5. The choice is structural rather than optimal: the mixture view motivates what information may be useful, but it does not prove that these nine projections are sufficient, minimal, or uniformly superior. A regularized logistic evaluator estimates their joint relevance from auxiliary episodes.

Source episodes create $H_{0}$ and $H_{1}$ examples by withholding identities, allowing the evaluator to learn without target unknowns. Leave-one-out gallery scores provide the accepted-known reference distribution for threshold calibration. The same fixed-dimensional profile also permits equal-budget pseudo episodes to compare candidate encoders after candidate-specific standardization rather than treating raw distance scales as comparable. Equations (5) and (6) do not guarantee separability: representation failure, gallery imbalance, or domain shift can remove the expected difference. The paired experiments and the nested profile-size analysis test whether the proposed evidence is useful in practice.

### 3.3. Nine-Statistic Gallery Profile

**Figure 4:**
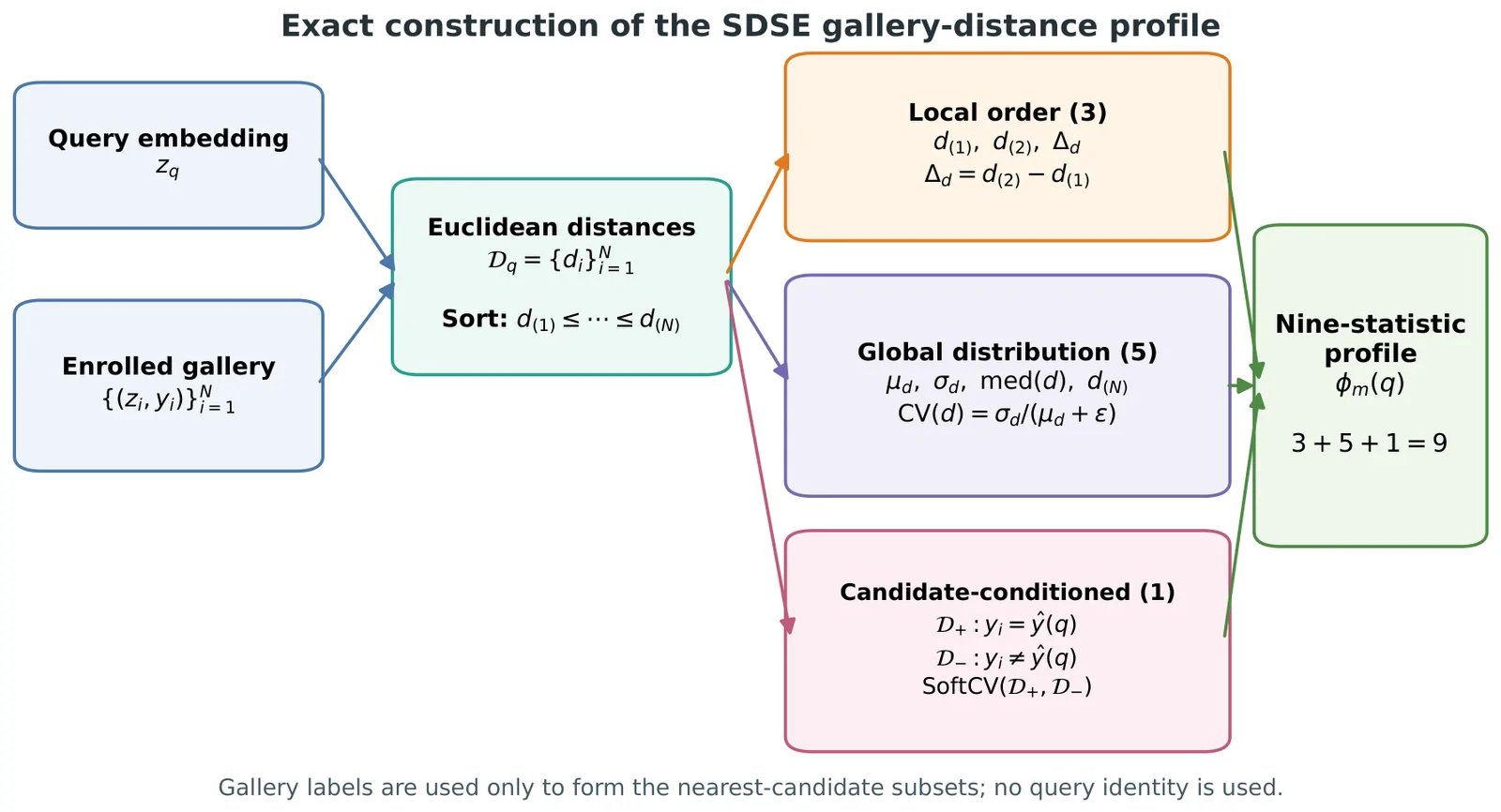
Construction of the nine-statistic gallery profile in Equation (7). The three local statistics are $d_{\left(1\right)}$, $d_{\left(2\right)}$, and their gap; the five global statistics are the mean, standard deviation, median, maximum, and CV; candidate-positive and candidate-negative dispersion produce SoftCV as the ninth statistic.

Figure 4 maps the distributional rationale in Table 5 to the nine computed quantities. Sort the distances as $d_{\left(1\right)}\leq \cdots \leq d_{\left(N\right)}$. The profile is

(7) $$\begin{array}{cc} \phi_{m}\left(q\right)=[\; & d_{\left(1\right)},d_{\left(2\right)},\mu_{d},\sigma_{d},\mathrm{med}\left(d\right),d_{\left(N\right)},\\ \; & \mathrm{CV}\left(d\right),\Delta_{d},\mathrm{SoftCV}\left(d\right)], \end{array}$$

where $\mathrm{CV}\left(d\right)=\sigma_{d}/\left(\mu_{d}+\epsilon \right)$ is the coefficient of variation, and $\Delta_{d}=d_{\left(2\right)}-d_{\left(1\right)}$. The first six terms retain absolute location and spread information, CV describes relative dispersion, and the gap indicates how strongly the nearest sample is separated from its closest competitor.

Let $\hat{y}\left(q\right)$ be the identity of the nearest gallery sample, and define

(8) $$D_{+}=\left\{d_{i}:y_{i}=\hat{y}\left(q\right)\right\},\;D_{-}=\left\{d_{i}:y_{i}\neq \hat{y}\left(q\right)\right\}.$$

The ninth component is

(9) $$\mathrm{SoftCV}\left(d\right)=\frac{\mathrm{CV}\left(D_{+}\right)-\mathrm{CV}\left(D_{-}\right)}{\mathrm{CV}\left(D_{+}\right)+\mathrm{CV}\left(D_{-}\right)+\epsilon }.$$

For nonnegative distances, SoftCV lies in [−1,1] up to the numerical stabilizer. Its sign indicates whether the nearest candidate identity is relatively more or less dispersed than the rest of the gallery. Gallery labels are available because the gallery represents enrolled people; no query identity is used in Equation (9).

The profile mixes absolute and relative evidence. The first six components retain the distance scale, whereas CV and SoftCV are scale-invariant when ϵ=0 and approximately invariant for the small stabilizer used in practice. SoftCV adds an interpretable identity-conditioned quantity unavailable from unlabeled global summaries, although Section 4.10 shows that its aggregate incremental effect is small. The implementation follows the nine-statistic specification introduced in our preprint; nine is not assumed to be a uniquely necessary dimension. Across candidate encoders, $\phi_{m}\left(q\right)$ acts as a deterministic adapter from a model-specific feature space to a common evidence space: no trainable projection, classifier head, or gradient update is attached to $f_{m}$. Computing the distances costs $O(Nd_{m})$ per query. The current implementation sorts them in O(NlogN) time; selection-based order statistics could reduce profile extraction to O(N).

### 3.4. Source-Episodic Evaluator Learning

For target dataset *t*, the remaining datasets form the auxiliary source set $S_{-t}$. Identity-level episodes reproduce the hypotheses in Equation (4): an episode contains a support gallery, known queries drawn from its identities, and pseudo-unknown queries drawn from disjoint identities. Profiles and binary labels from all episodes are pooled to fit one regularized logistic evaluator. The target dataset is excluded from fitting, and the resulting evaluator is applied without target-domain adaptation.

Let $E_{-t}$ be the source episodes, $u_{e,q}\in \left\{0,1\right\}$ the known/unknown episode label, and $\ell_{\mathrm{bal}}$ class-balanced logistic loss. The evaluator parameters are

(10) $$\psi_{m}^{\left(-t\right)}=\arg\min_{\psi }\frac{1}{|E_{-t}|}\sum_{e\in E_{-t}}\frac{1}{|Q_{e}|}\sum_{q\in Q_{e}}\ell_{\mathrm{bal}}\;\left(u_{e,q},\psi \left(\phi_{m}\left(q\right)\right)\right)+\lambda {∥\psi ∥}_{2}^{2}.$$

The target dataset is excluded from this objective. Thus, success cannot be explained by fitting its cameras, identities, or unknown-query prevalence. In the main experiment, each source dataset contributes 80 episodes. Every episode draws 40 support identities and 40 disjoint pseudo-unknown identities, with two support and two query images per selected identity. Known queries share identities with the support gallery; pseudo-unknown queries do not. Equation (7) converts both groups to fixed-dimensional vectors. A standardizer and class-balanced logistic regressor estimate

(11) $$s_{m}\left(q\right)=P_{\psi_{m}^{\left(-t\right)}}\left(\mathrm{unknown}|\phi_{m}\left(q\right)\right).$$

The auxiliary episodes train $\psi_{m}^{\left(-t\right)}$ but never update $f_{m}$. Because support and pseudo-unknown identities are disjoint within every episode, the labels correspond directly to the two hypotheses in Equation (4) rather than to memorized person identities. Each repetition resamples identities and images without replacement within the episode; identities may reappear in later episodes, so repetitions are independently resampled rather than globally disjoint. A deterministic seed makes the sequence reproducible. The fitted component is limited to the standardized logistic evaluator.

### 3.5. Target-Known-Only Calibration

Each enrolled gallery image is temporarily treated as a query against all remaining gallery images, producing leave-one-out scores $\left\{s_{m,i}^{\mathrm{LOO}}\right\}$. The default rejection threshold is

(12) $$\tau_{m}={\mathrm{Percentile}}_{95}{\left\{s_{m,i}^{\mathrm{LOO}}\right\}}_{i=1}^{N}.$$

A query is rejected when $s_{m}\left(q\right)>\tau_{m}$; otherwise, the original embedding-distance ranking is returned. Under approximate exchangeability between future known queries and the leave-one-out gallery scores, the 95th percentile targets an empirical known rejection rate near 5%. It is a calibration rule, not a distribution-free coverage guarantee, because repeated identities and camera effects create dependence. Target unknown labels are not used to fit the evaluator or set the threshold. AUROC and AUPR evaluate score ranking. OSCR combines unknown rejection with correct Rank-1 identification, where correctness uses the same cross-camera filtering as official ReID Rank-1 and mean average precision (mAP). The false-positive rate at 95% true-positive rate (FPR@95) is lower-is-better.

### 3.6. Source-Selected Unknown-Candidate Gate

UCG is asymmetric: it re-examines queries accepted by SDSE and may reject those with high auxiliary risk. It cannot recover a known query that SDSE has already rejected. Its first cue is the gallery-distance energy:

(13) $$E_{m}\left(q\right)=-\log\sum_{i=1}^{N}\exp\left[-d_{i}^{\left(m\right)}\left(q\right)\right].$$

This retrieval-space analogue of energy scoring requires no classifier logits. The second cue is the diagonal Mahalanobis deviation of the query embedding from the enrolled gallery:

(14) $$M_{m}\left(q\right)={\left[\sum_{j=1}^{d_{m}}\frac{{\left(z_{j}-\mu_{j}\right)}^{2}}{v_{j}+\epsilon }\right]}^{1/2},$$

where $\mu_{j}$ and $v_{j}$ are the gallery mean and variance in embedding dimension *j*. The diagonal form remains defined when the embedding dimension exceeds the number of enrolled samples and avoids an unstable covariance inverse.

Let $C_{E,m}$ and $C_{M,m}$ denote the corresponding known-gallery reference scores; self-distance is excluded when computing gallery energy. Each cue is placed on the scale of the gallery used in that split: a source gallery during weight search and the enrolled target gallery during deployment. The normalization is

(15) $${\tilde{r}}_{m}\left(q\right)=\frac{r_{m}\left(q\right)-\min C_{r,m}}{\max C_{r,m}-\min C_{r,m}+\epsilon },\;r\in \left\{E,M\right\}.$$

For α∈A={0,0.1,…,1}, the auxiliary risk is

(16) $$h_{m,\alpha }\left(q\right)=\alpha {\tilde{E}}_{m}\left(q\right)+\left(1-\alpha \right){\tilde{M}}_{m}\left(q\right).$$

The two labeled auxiliary datasets are each partitioned into enrolled and pseudo-unknown identities under the same disjoint query/gallery protocol used for evaluation. For every source proxy split, *r*, and candidate weight, α, the source-side threshold is computed only from its enrolled reference scores,

(17) $$\eta_{m,r,\alpha }^{\mathrm{src}}={\mathrm{Percentile}}_{95}\left\{h_{m,\alpha }\left(g_{i}\right):g_{i}\in G_{r}^{k}\right\},$$

and the corresponding known acceptance and pseudo-unknown rejection are evaluated using this threshold. Their means across the two source domains define ${\bar{A}}_{k}^{\mathrm{src}}$ and ${\bar{R}}_{u}^{\mathrm{src}}$. The energy weight is then selected without target queries:

(18) $$\alpha_{m}^{*}=\arg\max_{\alpha \in A}{\bar{R}}_{u}^{\mathrm{src}}\left(\alpha \right)\;s.t.\;{\bar{A}}_{k}^{\mathrm{src}}\left(\alpha \right)\geq 0.95,$$

Ties favor higher known acceptance and then higher candidate precision. If no weight satisfies the 0.95 constraint, the same lexicographic criterion is applied to all weights. After $\alpha_{m}^{*}$ is frozen, the target threshold is

(19) $$\eta_{m}={\mathrm{Percentile}}_{95}\left\{h_{m,\alpha_{m}^{*}}\left(g_{i}\right):g_{i}\in G^{k}\right\}.$$

With $b_{m}\left(q\right)=I\left[s_{m}\left(q\right)>\tau_{m}\right]$, the final decision is

(20) $$b_{m}^{\mathrm{UCG}}\left(q\right)=b_{m}\left(q\right)\;\vee \;I\left[h_{m,\alpha_{m}^{*}}\left(q\right)>\eta_{m}\right].$$

UCG changes only the binary operating decision. It is not applied when computing AUROC, AUPR, FPR@95, or OSCR, and it does not change the ReID ranking. Auxiliary labels are used to select the weight, while target calibration uses only the enrolled gallery. The Mahalanobis branch serves as an ablation of the two-cue hypothesis. When source-side selection returns α=1, that branch is inactive and can be omitted at inference.

### 3.7. Exploratory Frozen-Encoder Selection from the Enrolled Gallery

Assume a bank, $M=\{m_{1},\ldots ,m_{K}\}$, of already trained encoders. The nine-dimensional profile provides the same gallery-evidence interface for every candidate, while candidate-specific standardization handles distance-scale differences. This interface defines a possible selection protocol; its empirical validity must be established for the composition of the candidate bank and is not implied by dimensional compatibility alone. Selection compares proxy AUROC or OSCR, not raw distances, and must not inspect $Q^{k}$ or $Q^{u}$. For every candidate and target split, SDSE is fitted from the same auxiliary datasets using 40 fixed-budget source episodes with 30 support and 30 pseudo-unknown identities. The enrolled target gallery then generates 20 proxy episodes:

1. Sample at most 40 pseudo-known identities with cross-camera observations and at most 40 disjoint withheld identities.
2. Use two images per pseudo-known identity as support and one different-camera image as a known query.
3. Use two images per withheld identity as pseudo-unknown queries.
4. Average proxy AUROC and proxy OSCR across episodes.

The unknown-detection selector is

(21) $$m_{\mathrm{AUC}}^{*}=\arg\max_{m\in M}{\hat{\mathrm{AUROC}}}_{G}\left(m\right),$$

and the open-set-identification selector replaces the criterion with ${\hat{\mathrm{OSCR}}}_{G}\left(m\right)$. All candidates receive the same identity and episode budgets. Only after selection are official target queries evaluated. Selection regret for metric *J* is

(22) $$R_{J}=\max_{m\in M}J\left(m\right)-J\left(m_{J}^{*}\right).$$

Target-gallery identities are resampled independently across proxy repetitions, with pseudo-known and withheld pseudo-unknown sets disjoint within each episode but not forced to be disjoint across episodes. The seed–episode pair determines each split. The same identity and image indices are reused for every candidate in a dataset–seed trial, so model differences cannot be attributed to different proxy samples.

The procedure selects among existing frozen models; training the candidate bank is outside its scope. Task-aligned resampling supplies a testable proxy: if an encoder preserves cross-camera identity structure in the enrolled gallery, repeated withheld-identity episodes should predict its behavior on unseen queries. Correlation, top-1 agreement, and regret measure that hypothesis within the tested candidate bank.

### 3.8. End-to-End Procedure

The complete algorithm follows five stages, summarized visually in Figure 2 and Figure 3.

1. **Prepare a gallery interface.** Freeze each candidate encoder, compute query-to-gallery distances, and map every distance vector to the profile in Equation (7).
2. **Learn across auxiliary identity tasks.** For target dataset *t*, fit $\psi_{m}^{\left(-t\right)}$ with Equation (10); independently select the optional UCG weight with Equation (18).
3. **Select a candidate when needed.** Generate equal-budget pseudo-known and pseudo-unknown episodes from the enrolled target gallery and minimize the proxy regret in Equation (22). With a single encoder, this stage is skipped.
4. **Calibrate from enrolled target data.** Use leave-one-out gallery scores to set $\tau_{m}$ in Equation (12) and, if UCG is enabled, set $\eta_{m}$ in Equation (19).
5. **Infer without adaptation.** Compute $s_{m}\left(q\right)$ for a new query, apply Equation (20) at the selected operating point, and return the original cross-camera distance ranking only when the query is accepted.

This factorization is central to the design: source episodes learn a transferable presence rule, target-known data calibrate its operating point, and official target queries provide the final independent evaluation. No stage requires target unknown labels or a gradient update to the ReID encoder.

## 4. Experiments

### 4.1. Datasets, Embeddings, and Protocol

We evaluate Market-1501, DukeMTMC-reID, and CUHK03 with their official disjoint query/gallery partitions [23,24,25]. The partitions encode six, eight, and two cameras, respectively. For each dataset, 50% of test identities are sampled as enrolled; they retain their gallery images and contribute known queries. The remaining identities contribute unknown queries only. The primary seven-method comparison uses seeds 1–5, whereas the confirmatory SDSE–nearest comparison uses seeds 1–20. Within every paired comparison, all scorers share the encoder, gallery, queries, and identity split. Table 6 reports the resulting gallery and query composition.

CUHK03 uses its labeled new-protocol query/gallery split. Exported feature archives retain the image path, identity, camera, and split flag for each row; the public scripts verify this alignment before producing qualitative retrieval results.

The main comparison uses a 512-dimensional OSNet-x1.0 initialized from ImageNet and trained for 60 epochs on Market-1501. Training images are resized to 256×128 and randomly flipped horizontally. The batch contains 64 images, eight per identity. Adam uses a learning rate of $3\times 10^{-5}$ and weight decay $5\times 10^{-4}$. Label-smoothed identity classification and triplet loss receive equal weight, with triplet margin 0.3. The encoder uses training seed 1; the open-set identity splits use seeds 1–5 for the primary comparison and 1–20 for confirmation. Euclidean scorers operate on exported pre-classifier vectors without additional $L_{2}$ normalization. Market-1501 is, therefore, the in-domain representation reference, while DukeMTMC-reID and CUHK03 test transfer to different camera systems.

Rank-1 and mAP follow the official cross-camera filtering rules, and OSCR uses exactly the same filtered Rank-1 correctness. Known queries without a valid cross-camera positive are excluded from the retrieval and OSCR denominators. Unknown scores are computed for every query because a deployed system cannot use query identity to decide whether a camera filter should apply. This common protocol keeps unknown detection, known retrieval, and combined open-set identification distinct but compatible.

The baseline set comprises energy, unit-normalized cosine distance (1− maximum cosine similarity), nearest Euclidean distance, mean 5-nearest-neighbor distance, novelty-mode local outlier factor (LOF, k=20), and shrinkage Mahalanobis distance with Ledoit–Wolf covariance. Whenever fitting is required, it uses enrolled gallery embeddings only. Hyperparameters k=5 and k=20 remain fixed across datasets. The SDSE standardizer is fitted on auxiliary profiles; its class-balanced logistic regressor uses an $L_{2}$ penalty, C=1, L-BFGS optimization, and at most 1000 iterations. For each target and seed, UCG compares the 11 weights in A on the two auxiliary source-domain proxy splits, never on official target queries.

Primary results are means and sample standard deviations over five paired seeds. Confirmatory inference first macro-averages the three datasets within each of 20 seeds and then reports the mean paired difference, a 95% percentile bootstrap interval from 20,000 resamples, and a two-sided Wilcoxon signed-rank test. FPR@95 differences are sign-reversed so that every positive effect favors SDSE. This paired design controls identity-partition variability and avoids treating dataset-level measurements from the same split as independent observations.

The main SDSE experiment uses 80 source episodes per auxiliary dataset with 40 enrolled and 40 pseudo-unknown identities because it provides the final query-level score. The encoder-selection sweep repeats fitting for six candidates, three targets, and five seeds; it, therefore, uses a fixed 40-episode source budget with 30 enrolled and 30 pseudo-unknown identities, followed by 20 target-gallery proxy episodes. This reduced budget controls the repeated computational cost and is applied identically to every candidate. The selection experiment is a ranking study within the candidate bank, not a replacement estimate of the main 80-episode SDSE result. Source and target-proxy identities are resampled independently across repetitions, with disjoint known/pseudo-unknown sets only within an episode. For a given target proxy episode, the sampled identities and image indices are identical across all candidate encoders. All candidate features are exported from the same test images after the same deterministic preprocessing: resize to 256×128, tensor conversion, and channel normalization by ImageNet mean (0.485,0.456,0.406) and standard deviation (0.229,0.224,0.225). Thus, preprocessing does not vary with the selected encoder.

Gallery sensitivity is evaluated separately over seeds 1–5. Starting from the same 50% known split, we test nested 25%, 50%, and 100% subsets of the enrolled identities; one image, at most two images, and all available images per enrolled identity; a balanced one-image gallery and an induced long tail in which alternating identities retain one or all images; and unknown-query proportions of 25%, 50%, and 75%. The evaluator is not refitted for any target-gallery condition. To equalize computation and class support, sensitivity trials use at most 400 known and 400 unknown official queries per dataset–seed; the main and 20-split comparisons continue to use every official query.

The experiments test four linked hypotheses. First, a distributional profile should improve unknown-risk ranking when the nearest extreme is poorly calibrated. Second, any gain should persist under controlled gallery perturbations but cannot replace missing same-identity evidence. Third, open-set identification should remain bounded by frozen-encoder retrieval. Fourth, withheld-identity gallery episodes should provide an informative proxy for ranking frozen encoders. The result sections follow this order, keeping score, representation, threshold, and selection effects identifiable.

### 4.2. Open-Set Scoring Results

Table 7 reports the macro average across datasets. We first average the three datasets within each seed and then compute the mean and standard deviation over the five seeds. Relative to nearest distance, SDSE gains 0.0092 AUROC, 0.0087 AUPR, and 0.0038 OSCR, and lowers FPR@95 by 0.0192.

The baseline suite isolates three competing explanations. Mean 5-NN distance tests whether averaging the local tail is sufficient; LOF tests local density; shrinkage Mahalanobis scoring tests global embedding deviation. SDSE achieves the highest macro AUROC and AUPR among the evaluated frozen-embedding scorers, indicating that its advantage is not explained by any one of these alternatives. OpenMax and PROSER are methodologically adjacent but require a fixed parametric classifier interface or classifier retraining [13,14]. They are, therefore, compared conceptually in Section 2.2 rather than converted into different algorithms for the replaceable-gallery setting.

**Figure 5:**
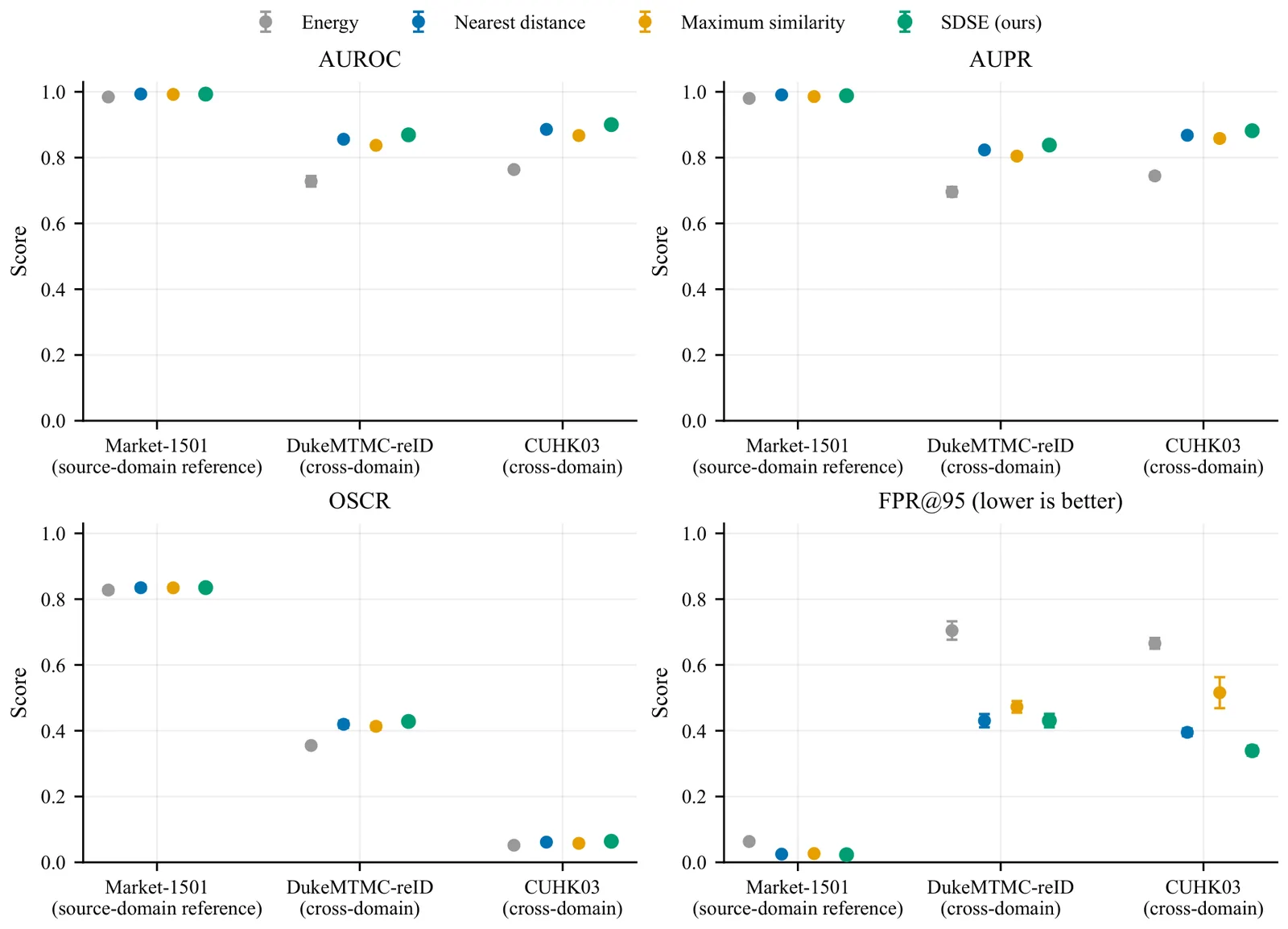
Five-split results for four core scorers on the Market-1501 representation-training reference and two shifted camera domains. Markers show means and bars show one sample standard deviation; Table 7 reports the complete seven-method comparison. FPR@95 is lower-is-better.

Table 8 and Figure 5 reveal where the macro gain arises. On Market-1501, the training-domain encoder already gives nearest distance an AUROC of 0.993; SDSE is effectively tied and nearest distance retains a small AUPR advantage. On DukeMTMC-reID and CUHK03, the nearest extreme is less stable under transfer, and the additional distributional evidence raises AUROC, AUPR, and OSCR in every split. CUHK03 FPR@95 decreases from 0.395 to 0.339, while DukeMTMC-reID shows no corresponding FPR@95 improvement. The scientific conclusion is conditional: the profile contributes when domain transfer weakens nearest-distance calibration, not when that statistic is already near the ceiling.

### 4.3. Qualitative Query–Gallery Analysis

**Figure 6:**
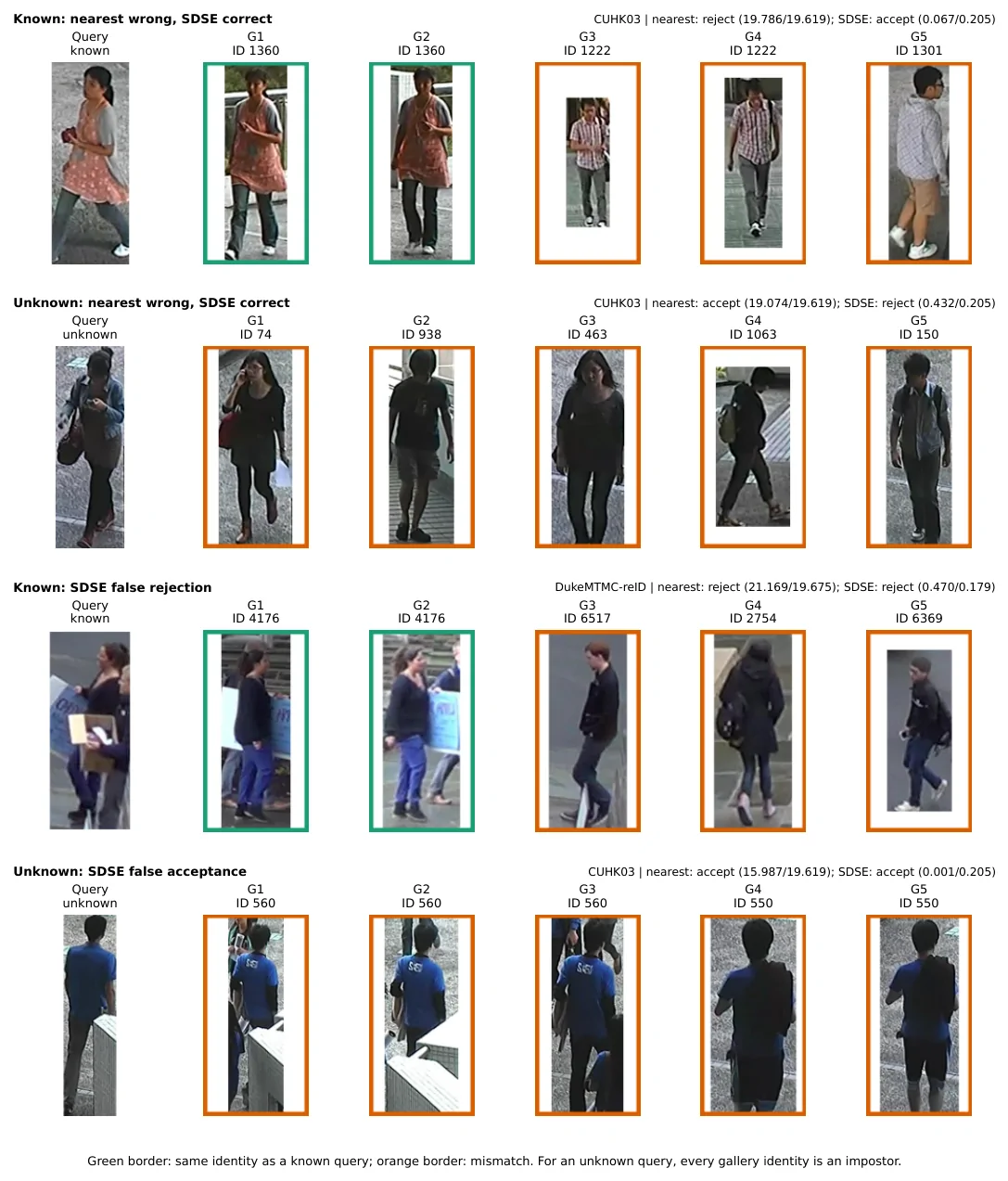
Representative Query–Gallery decisions under the same seed-1 protocol as the quantitative evaluation. Each row shows the query followed by its top-five gallery images after official same-identity/same-camera filtering for known-query display. Green borders mark the query identity; orange borders mark mismatches. Every gallery image is necessarily an impostor for an unknown query. Scores are shown as value/target-known P95 threshold, and larger values indicate greater unknown risk. Rows 1–2 show a known false rejection and an unknown false acceptance by nearest-distance thresholding that SDSE corrects. Rows 3–4 expose an SDSE false rejection and false acceptance, respectively.

Figure 6 connects the score-level results to the retrieval decisions seen by a camera-network operator. The four rows are selected deterministically from seed 1 after all query scores and target-known thresholds have been fixed: the first two maximize the normalized decision-margin change among cases corrected by SDSE, and the last two show the corresponding highest-margin SDSE errors. Ground-truth unknown labels are used only at this final visualization stage to assign a scored query to a case category; they do not enter evaluator learning, threshold calibration, or quantitative model selection.

The corrected known case retains valid cross-camera matches at ranks 1–2 even though its absolute nearest distance crosses the baseline threshold; SDSE accepts it because the complete gallery profile remains compatible with an enrolled identity. In the corrected unknown case, a deceptively close impostor passes the nearest-distance threshold, whereas the broader profile triggers rejection. The failure rows delimit the same mechanism: a shifted known profile can still be rejected despite correct ranking, and an unknown person with several gallery look-alikes can resemble a known profile. These examples explain the decision layer but do not replace the paired population-level evidence in Table 7, Table 8 and Table 9.

### 4.4. Paired Effects and Split Stability

**Figure 7:**
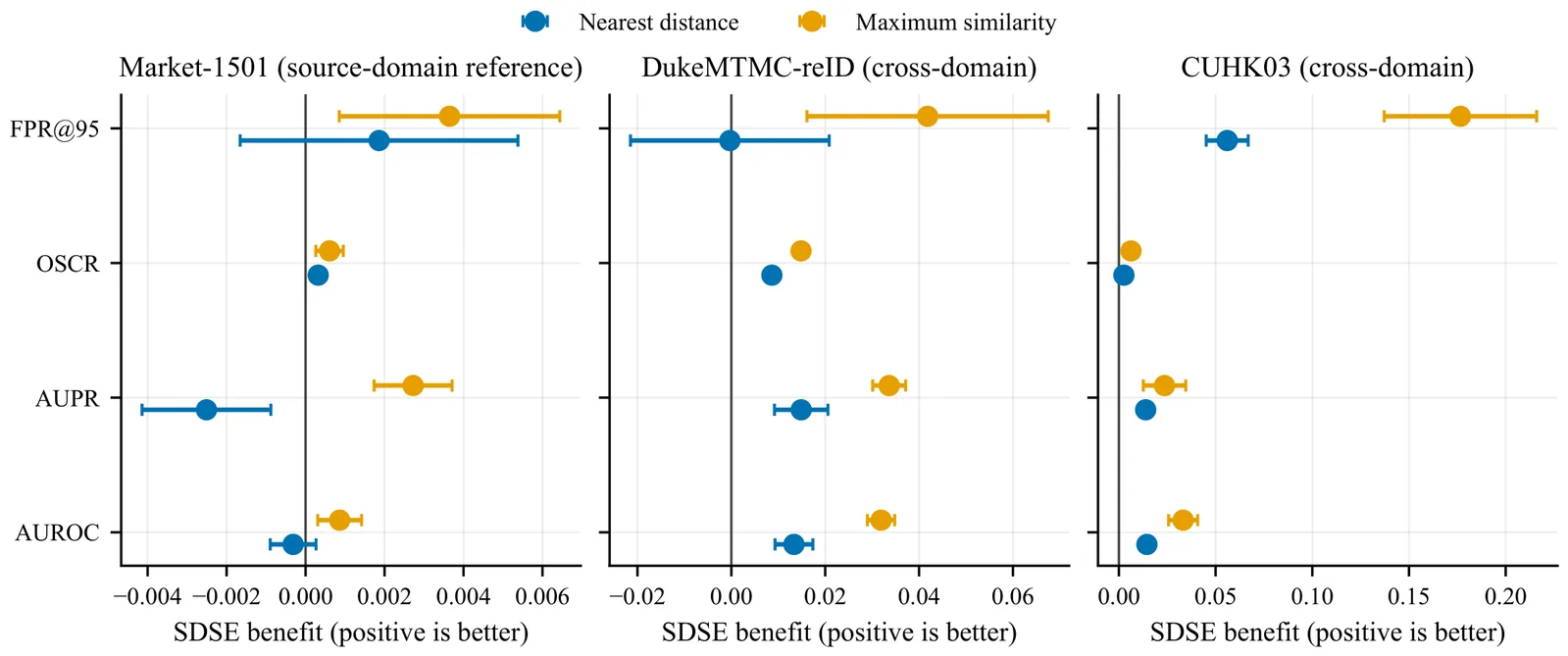
Paired SDSE benefit on identical identity splits. For FPR@95, the sign is reversed so positive values always denote improvement.

Figure 7 presents the primary five-split effect sizes and orients every difference so that positive favors SDSE. Against nearest distance, DukeMTMC-reID gains are +0.0133 AUROC, +0.0149 AUPR, and +0.0086 OSCR. CUHK03 gains are +0.0145 AUROC, +0.0138 AUPR, and +0.0026 OSCR, with FPR@95 lower by 0.0560. Market-1501 differences are negligible in AUROC and OSCR, while its AUPR favors nearest distance by 0.0025. Inferential claims are based on the larger paired analysis in Table 9, not on five observations.

**Figure 8:**
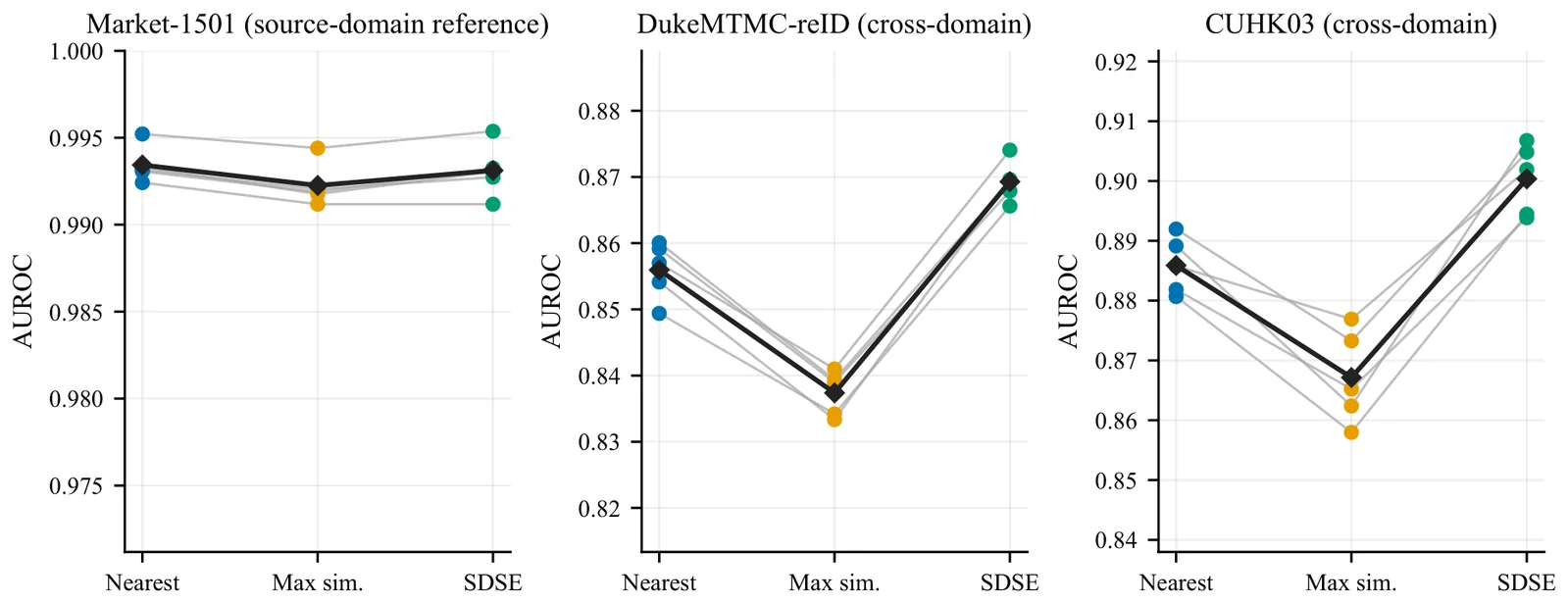
AUROC for each known/unknown identity-split seed. Blue, orange, and green markers denote nearest distance, maximum similarity, and SDSE, respectively; thin gray lines connect scores evaluated on the same split, and black diamonds joined by a black line denote method means.

Figure 8 shows the AUROC ordering for every primary seed. SDSE exceeds nearest distance and maximum similarity on both transferred datasets in all five splits, so the displayed gain is not driven by a single favorable partition.

All four intervals exclude zero, and every paired difference favors SDSE. The magnitude remains important: AUROC and AUPR improve by about one percentage point, whereas OSCR improves by only 0.00365 because a known query must be both accepted and retrieved correctly. Table 10 connects this central effect to the evidence for each additional design component.

### 4.5. Gallery-Composition Sensitivity

Table 11 tests whether the distributional advantage survives changes to the amount and balance of gallery evidence.

SDSE retains a positive AUROC and OSCR difference from the nearest distance in every controlled condition. Across small, medium, and large identity galleries, the AUROC gain remains between 0.0098 and 0.0120. Absolute performance, however, depends strongly on gallery evidence: reducing the gallery from all available images to one image per identity lowers mean SDSE AUROC from 0.9182 to 0.7078 and Rank-1 from 0.449 to 0.331. The induced long tail is also harder than the full gallery despite a positive relative gain. These results agree with the mixture rationale: the evaluator can interpret a same-identity component when evidence exists, but it cannot reconstruct missing reference observations. Varying unknown prevalence leaves AUROC and OSCR nearly stable while changing AUPR substantially, as expected from the prevalence dependence of precision–recall.

### 4.6. Exploratory Target-Unknown-Free Frozen-Encoder Selection

The candidate bank contains six frozen representations: an ImageNet-initialized ResNet50-AE feature extractor, an ImageNet-pretrained OSNet-x1.0, and four Market-1501 OSNet-x1.0 checkpoints saved after 10, 30, 60, and 80 epochs. The bank varies initialization and training stage but contains only two architecture families and is dominated by one ReID source domain. It, therefore, tests within-bank feasibility rather than transfer across unrelated architectures, self-supervised objectives, or source domains. No candidate is trained or fine-tuned during selection.

Table 12 evaluates the selector defined in Section 3.7. Spearman correlation is computed across the six candidates in each dataset-seed trial and then averaged over seeds. Top-1 hit records exact agreement with the oracle chosen from official target-query performance, while regret measures the cost of a different choice. For AUROC, mean Spearman correlation ranges from 0.943 to 0.977 and mean regret from 0.00027 to 0.00264. For OSCR, the corresponding correlations range from 0.886 to 0.977. Market-1501 has the largest mean OSCR regret because its candidates have nearly identical unknown-detection scores but small differences in retrieval-coupled OSCR.

**Figure 9:**
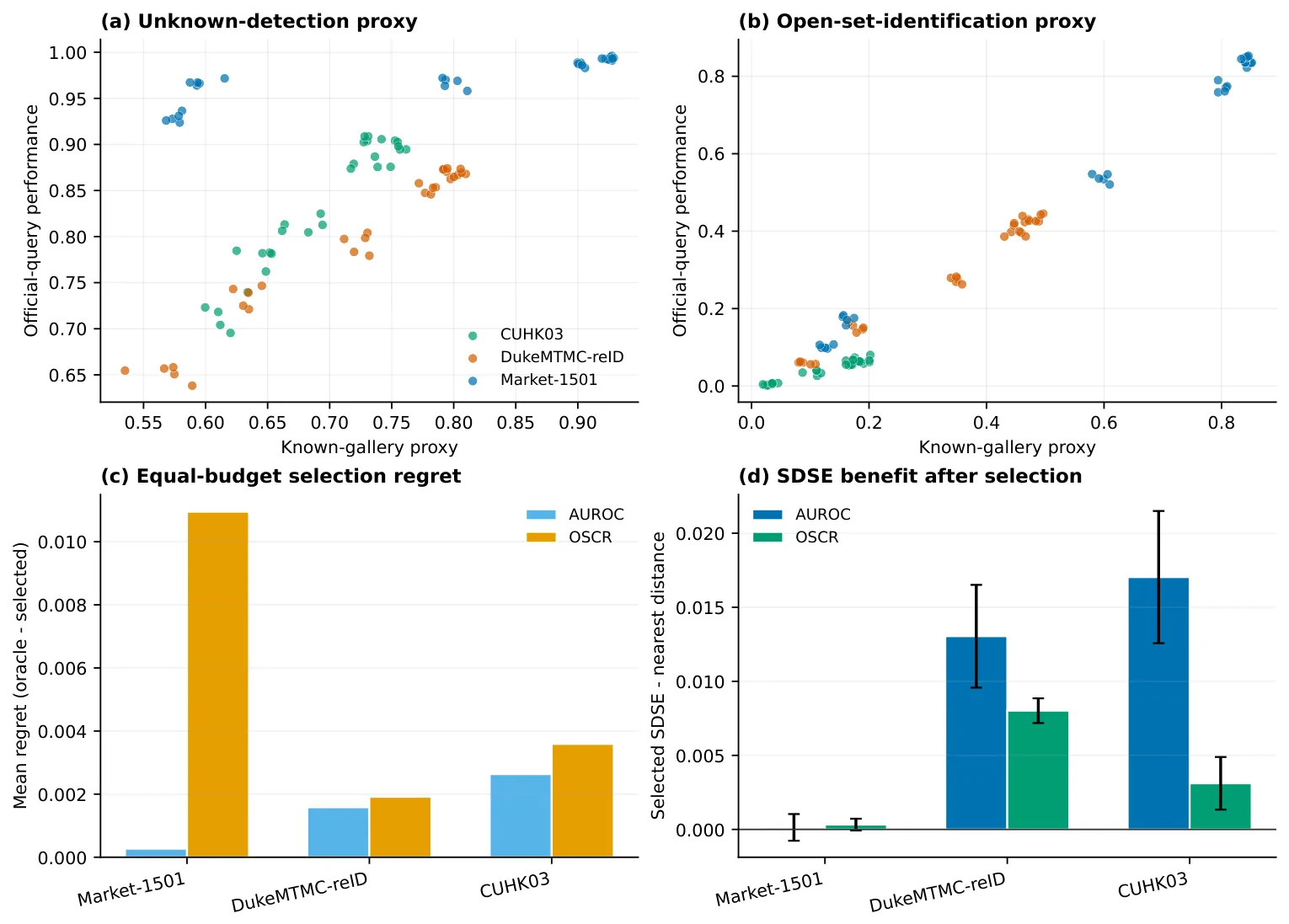
Validation of target-gallery model selection across six frozen encoders and five seeds. (**a**,**b**) Gallery-only proxies versus official-query performance. (**c**) Mean regret relative to an oracle that sees official target queries. (**d**) SDSE improvement over nearest distance after proxy-based encoder selection.

Across the 15 dataset–seed trials, mean regret is 0.00150 for AUROC selection and 0.00549 for OSCR selection. The selector exactly matches the oracle in 53.3% and 40.0% of trials, respectively; low regret despite a top-1 mismatch indicates that several candidates can be practically equivalent. Figure 9 visualizes the proxy–oracle relationship and shows that the gallery episodes preserve the candidate ordering more reliably than exact winner identity. The experiment, therefore, supports task-aligned ranking within this bank. Its four checkpoints from one OSNet training run, however, do not test architecture-general selection.

### 4.7. Representation Boundary and Evaluator Capacity

Unknown scoring cannot compensate for a representation that fails to retrieve enrolled identities. Table 13 reports known-query performance for the 60-epoch OSNet embeddings. Because SDSE changes only the acceptance score, OSCR remains bounded by the Rank-1 accuracy of the underlying representation.

At every OSCR threshold, the correctly accepted fraction cannot exceed the Rank-1 fraction of evaluable known queries; integrating over thresholds preserves this upper bound. On CUHK03, the transferred encoder obtains Rank-1 0.066±0.008, and SDSE obtains OSCR 0.064±0.007. The low OSCR is, therefore, a representation ceiling, not a protocol inconsistency: both quantities use the same official cross-camera filtering.

CUHK03, therefore, separates the three layers of evidence. AUROC 0.900 shows that the profile orders unknown risk effectively; Rank-1 0.066 shows that the frozen representation rarely retrieves the correct enrolled identity; OSCR 0.064 combines both requirements and approaches the retrieval ceiling. The dataset supports the unknown-person detector but not strong end-to-end identification with this transferred encoder. This distinction also explains why improving the post hoc score cannot repair a weak representation.

On seed 1, replacing logistic regression with Extra Trees or gradient boosting yields three-dataset macro AUROC values of 0.899 and 0.909, compared with 0.921 for logistic regression. Their macro OSCR values are 0.427 and 0.431, compared with 0.436. The higher-capacity evaluators provide no advantage in this experiment, supporting the use of a simple, well-regularized decision model.

### 4.8. Adaptive False-Accept Correction

Table 14 evaluates the decision rule from Section 3.6. Adaptive UCG raises macro unknown rejection from 0.380 to 0.472 while retaining 0.946 known acceptance. Queries flagged by the gate have precision 0.865, and the decision changes for only 5.82% of all queries. The benefit is accompanied by the expected cost: some difficult known queries are also rejected.

The selected weights identify the operative mechanism. Source-constrained search chooses α=1 in 14 of the 15 dataset–seed trials and α=0.8 once. Adaptive UCG and the fixed energy gate consequently have the same mean unknown rejection and known acceptance, while adaptive UCG has slightly lower gate recall. Source-side search contributes defensively by suppressing an ineffective cue without inspecting target unknowns; it does not improve on fixed energy in these experiments. Because the continuous SDSE score and all model parameters remain unchanged, Table 14 measures a threshold-level intervention rather than a new representation or scoring gain.

For the evaluated setting, the parsimonious deployment rule is, therefore, the energy-only gate. The Mahalanobis row functions as a negative control for the proposed two-cue hypothesis, and the branch can be omitted whenever α=1. This result is scientifically useful because it locates the improvement in energy-based threshold control rather than in unsupported cue synergy.

### 4.9. Profile Diagnostics and Threshold Calibration

**Figure 10:**
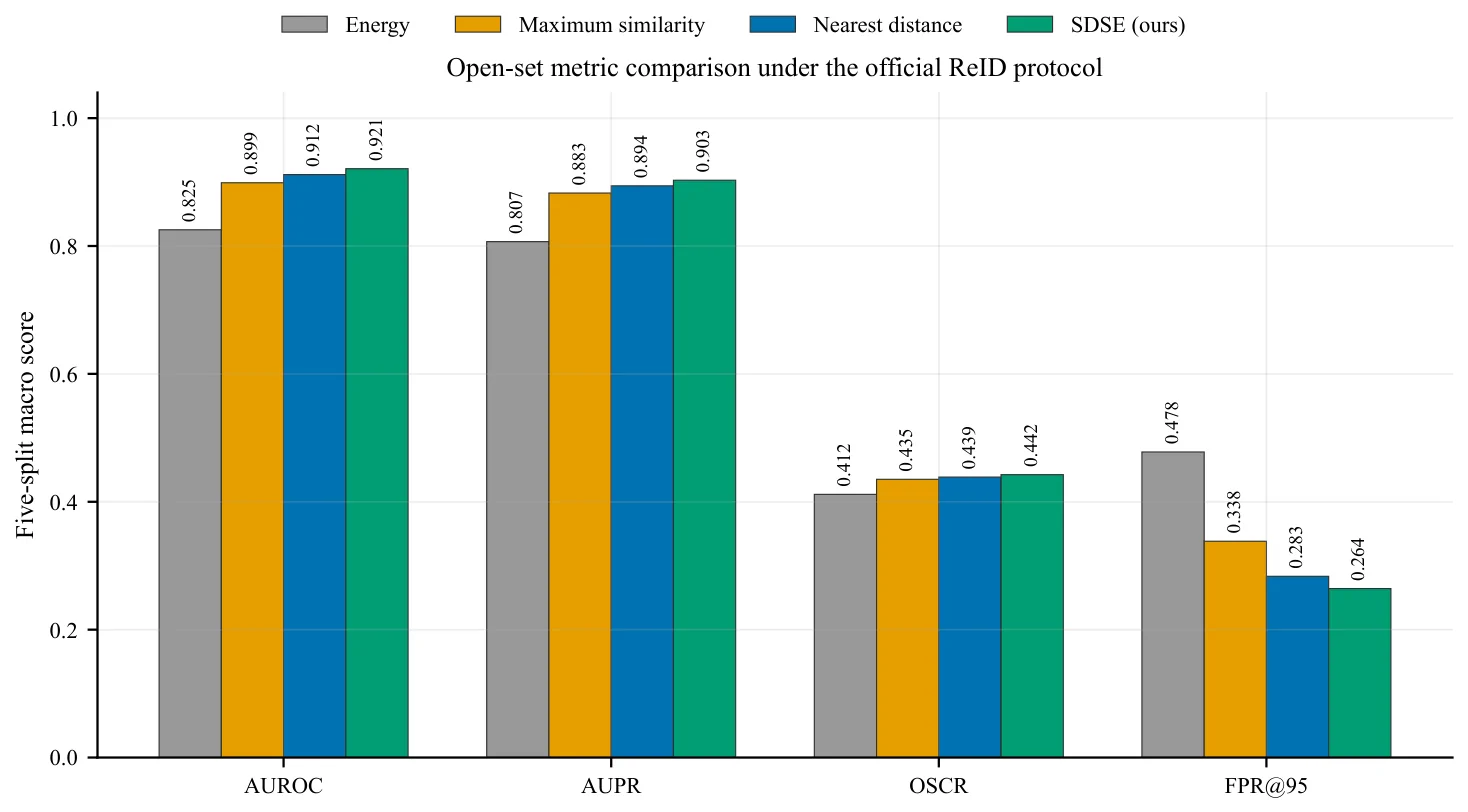
Selected core-scorer comparison over five splits; the complete seven-method results appear in Table 7. The difference between AUROC and OSCR reflects OSCR’s dependence on correct known-person retrieval.

**Figure 11:**
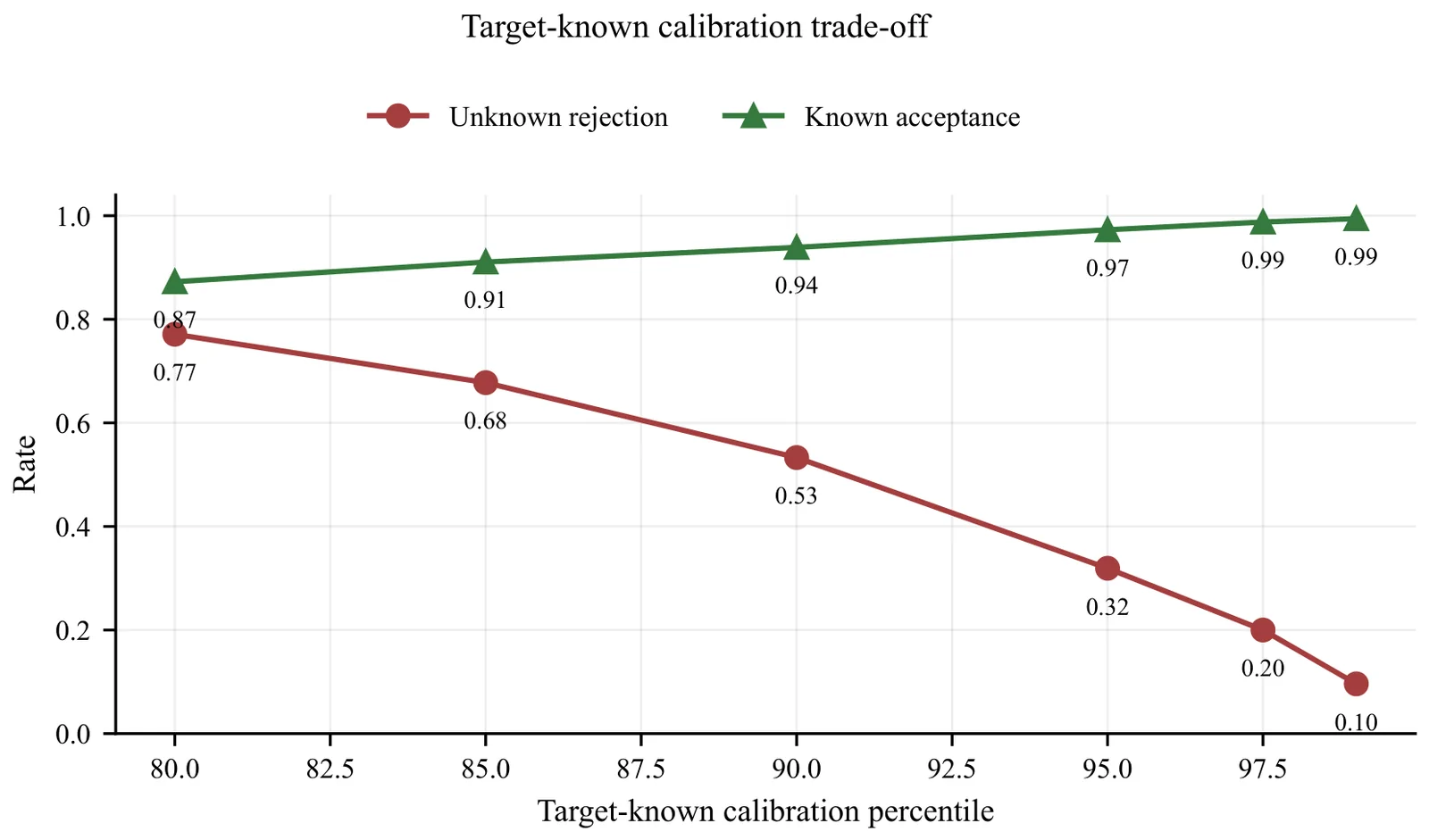
Threshold-calibration trade-off for a frozen SDSE detector. Seed 1 is macro-averaged across three datasets. Only the operating threshold applied to the frozen SDSE score changes; neither the ReID encoder nor the SDSE parameters are updated. Lower known acceptance reflects a decision-threshold trade-off rather than a change in representation quality.

**Figure 12:**
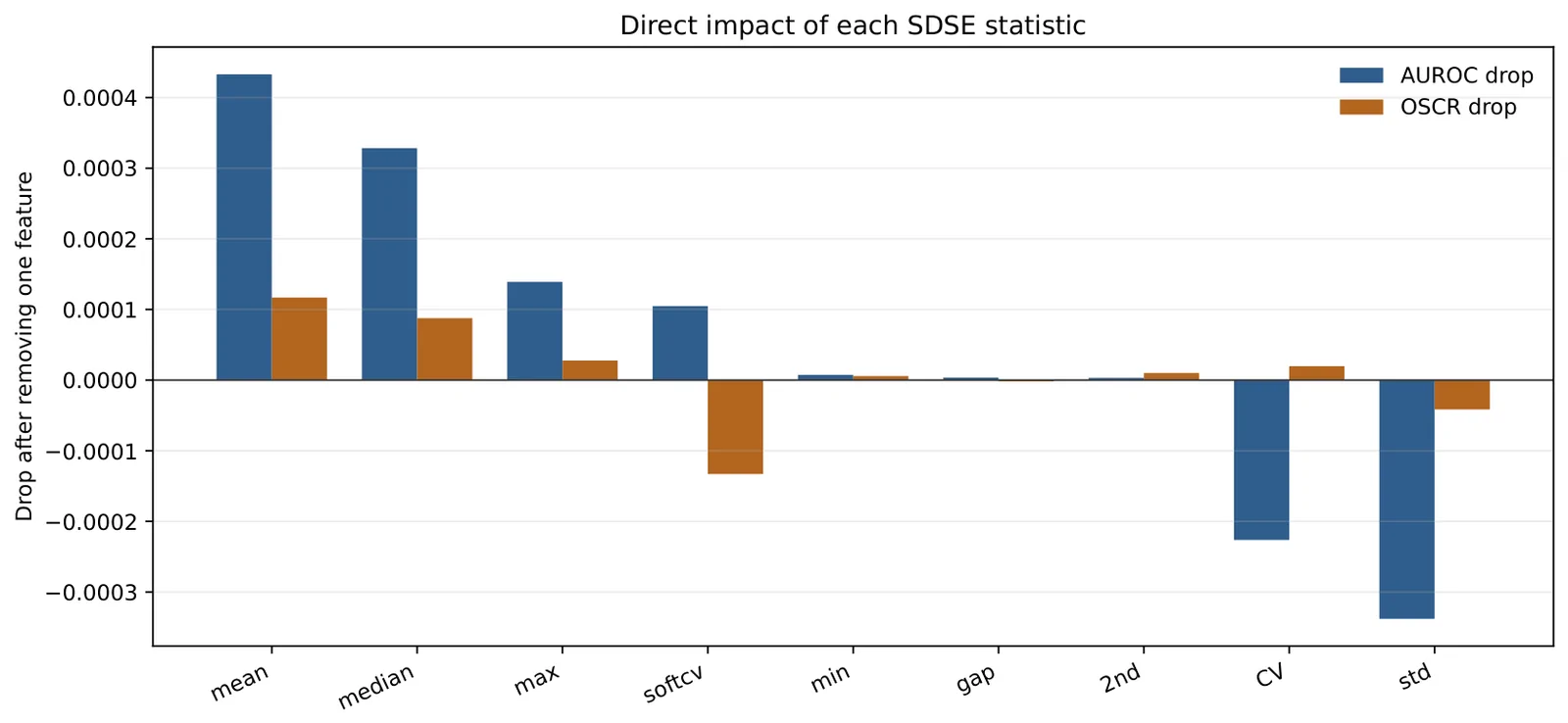
Leave-one-feature-out analysis of the nine SDSE statistics over five seeds. Positive values denote a performance decrease after removing the indicated statistic. Individual features are partly redundant; the experiment evaluates the joint profile rather than attributing the full gain to one component.

**Figure 13:**
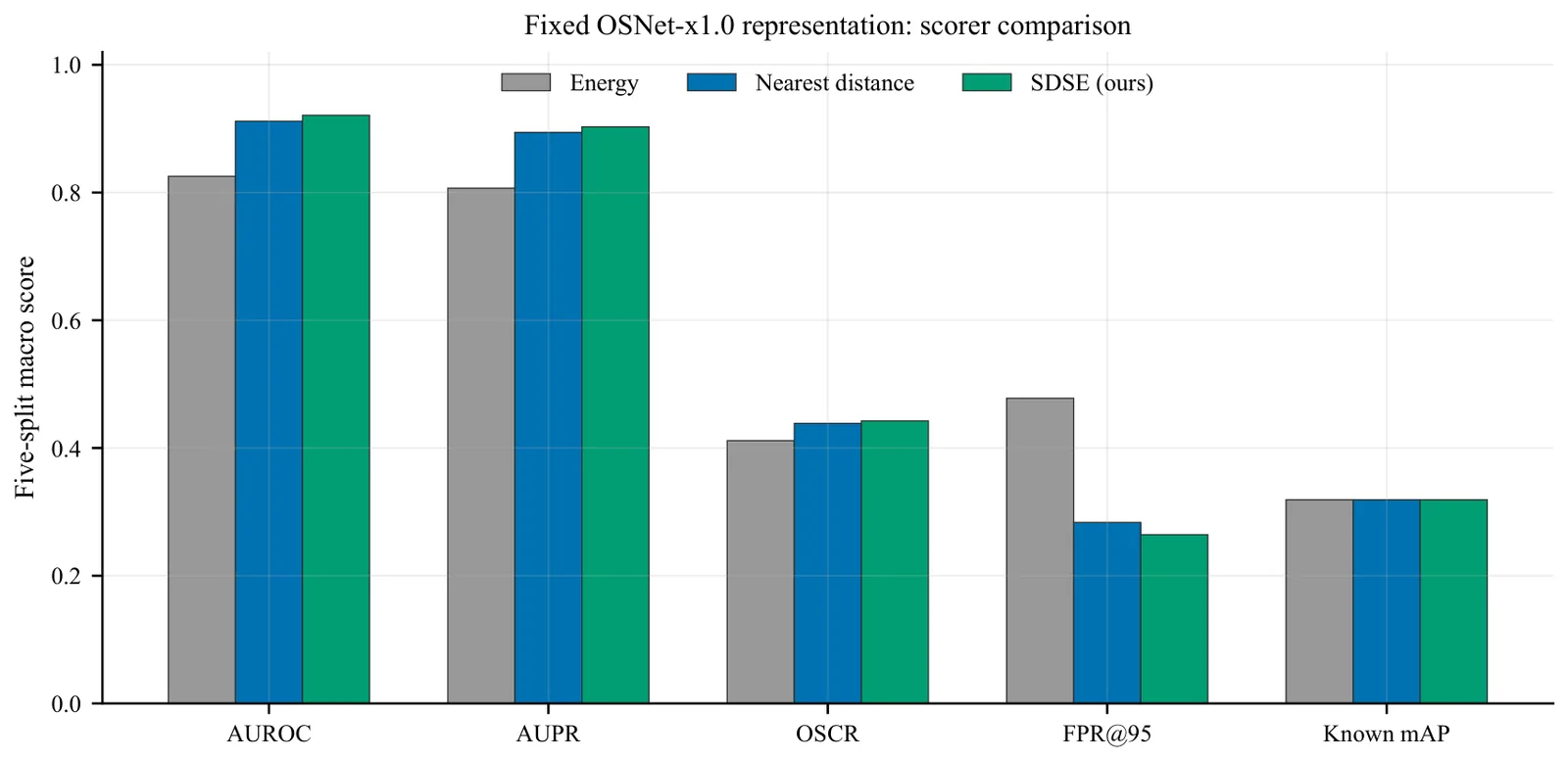
Fixed-embedding scorer comparison. Known mAP is shared because all scorers use the same OSNet-x1.0 ranking; score calibration cannot exceed the retrieval capacity of the representation.

Figure 10 compares the four open-set metrics, Figure 11 traces the calibrated operating-point trade-off, Figure 12 tests the contribution of each profile component, and Figure 13 compares scorers on fixed embeddings. All four analyses use the same official cross-camera correctness rule as Table 7 and Table 8.

The four diagnostics answer different questions about the decision layer. Figure 10 confirms that the main advantage lies in unknown-score separation, whereas the smaller OSCR change reflects the additional requirement of correct known-person retrieval. Figure 11 shows the expected exchange between unknown rejection and known acceptance when only the threshold changes. The leave-one-feature-out results in Figure 12 indicate that the profile is distributed and partly redundant rather than being driven by a single statistic. Finally, Figure 13 keeps the embedding fixed and, therefore, separates score calibration from representation quality: all scorers inherit the same known-query mAP.

### 4.10. Nested Profile-Size Analysis

Table 15 complements leave-one-feature-out deletion by adding evidence groups cumulatively. Local order statistics improve only slightly over nearest distance, and global summaries alone are insufficient. Combining the two raises macro AUROC from 0.9139 to 0.9208; the full profile reaches 0.9209. The result isolates the main principle: local match evidence becomes more informative when interpreted relative to the global gallery distribution. SoftCV remains an interpretable candidate-conditioned diagnostic, but the negligible eight-to-nine-statistic difference shows that it is not required for the aggregate gain.

### 4.11. Limitations

The nine statistics are designed projections of an empirical distribution, not sufficient statistics derived from a complete generative model. Equations (5) and (6) motivate their roles, while the nested analysis shows that the first eight match the full profile in aggregate. Gallery perturbations establish robustness only over the tested range; one or two reference images cause substantial absolute degradation, and arbitrary gallery growth, severe camera imbalance, and temporal change remain open. The evaluator also requires labeled auxiliary identities, even though target unknown labels and encoder adaptation are unnecessary.

The numerical baseline comparison is intentionally restricted to post hoc scorers that share the frozen, replaceable-gallery interface; classifier-dependent OpenMax and PROSER answer a different intervention question. Near-ceiling nearest-distance separation on Market-1501 leaves little room for SDSE. UCG reduces to energy in 14 of 15 trials, and the selector is evaluated on only six candidates from two architecture families. Low CUHK03 Rank-1 accuracy constrains OSCR independently of score quality. Finally, the experiments assume a static gallery and do not address camera drift, temporal association, discovery, or continual enrollment.

## 5. Conclusions

This study asked whether a complete query-to-gallery distance distribution can provide stronger identity-presence evidence than its nearest value when the ReID encoder is fixed. SDSE answers that question by combining local order, global distribution, and candidate-conditioned summaries within a source-episodic evaluator. Identity-disjoint auxiliary episodes supply the evaluator’s training examples, while target-known leave-one-out calibration fixes the operating point without target unknown labels. Its meta-validation structure transfers the decision rule across identity tasks and camera domains, while target-known leave-one-out calibration fixes the operating point without target unknown labels. On shared embeddings and paired identity splits, SDSE provides the strongest macro unknown detection among seven post hoc scorers; the 20-split analysis confirms a 0.0095 AUROC gain over nearest distance. The gain concentrates on the transferred DukeMTMC-reID and CUHK03 domains, where the nearest extreme is less reliable, and disappears on the near-ceiling Market-1501 reference. Profile-size and gallery-sensitivity experiments explain both the mechanism and its boundary: local evidence benefits from global context, but no decision layer can replace sparse same-identity references or weak retrieval. UCG further identifies energy as the useful threshold-control cue, while the gallery-only selector demonstrates low-regret ranking within a limited candidate bank. The resulting contribution is a scientifically grounded, lightweight decision layer that extends frozen ReID systems to fixed-gallery open-set operation while preserving their representations and accepted-query rankings. Broader encoder banks, dynamic galleries, temporal drift, and continual enrollment remain the next validation frontier. 

## Figures and Tables

**Table 1:** Relationship between the 2023 arXiv preprint and the present study.

Aspect	2023 Preprint [4]	Present Study
Problem formulation	Broad open-world learning illustrated with ReID data	Gallery-relative open-set ReID with explicit known/unknown hypotheses and cross-camera identification correctness
Nine statistics	Introduced the meta-characteristic representation and candidate-positive/candidate-negative CV contrast	Organizes the statistics into local, global, and candidate-conditioned evidence and tests grouped and nested profile sizes
Learned decision rule	No source-episodic, gallery-relative probability evaluator	Standardized, class-balanced logistic evaluator learned from auxiliary identity episodes while the encoder remains frozen
Deployment protocol	No target-known-only calibration or official ReID evaluation chain	Leave-one-out target-gallery calibration, official cross-camera Rank-1/mAP and OSCR, five paired identity splits, and post hoc novelty baselines
Additional analyses	Not included	UCG operating-point analysis and target-gallery frozen-encoder selection, evaluated separately from the main score

**Table 2:** Representative open-set and open-world ReID formulations. “Updated object” identifies what must change beyond ordinary gallery retrieval.

Work	Primary Open-World Function	Updated Object	Gallery Regime	Difference from the Current Setting
Liao et al. [8]	Probe-presence detection plus identification	Independent metric learner and operating threshold	Static	Establishes the task and protocol; does not learn a gallery-profile evaluator or select encoders
Li et al. [9]	Watch-list verification against similar impostors	Generator, feature extractor, and target discriminator	Static	Trains an adversarial open-world representation
Yu et al. [10]	Uncertainty-aware verification and retrieval	Distribution-valued ReID representation	Static	Retrains the representation to model sample uncertainty
Casao et al. [11]	Unknown discovery and incremental enrollment	Identity clusters and gallery models	Dynamic	Expands the gallery online rather than preserving a fixed enrollment set
Current work	Gallery-relative rejection with operating-point and model-selection analyses	Low-capacity profile evaluator; encoder frozen	Static	Preserves accepted-query ranking; evaluates an auxiliary gate and six-candidate selection

**Table 3:** Interpretation of the evaluation quantities. “Unknown” is the positive class for AUROC, AUPR, and FPR@95.

Quantity	Question Answered	Threshold Dependence	Dependence on ReID Correctness
AUROC	Does the score rank unknown queries above known queries?	Threshold-free	None
AUPR	How precise is unknown detection across recalls at the observed unknown prevalence?	Threshold-free	None
FPR@95	What known-query false-positive rate is required for 95% unknown recall?	One ROC operating point	None
Rank-1/mAP	Does the frozen encoder retrieve the enrolled identity?	No rejection threshold	Direct retrieval measures
OSCR	Can the system reject unknowns while accepting and correctly identifying knowns?	Integrated over score thresholds	Direct; OSCR is upper-bounded by Rank-1
Known acceptance/unknown rejection	What happens at the calibrated deployment threshold?	Threshold-specific	Acceptance does not imply a correct match

**Table 4:** Comparison with adjacent recognition and deployment settings. The updated component indicates the usual intervention in each setting.

Problem	Primary Uncertainty	Updated Component	Deployment Output
Cross-domain ReID	Camera/domain shift for matchable identities	Encoder or adaptation module	Ranked gallery identities
Classifier open-set/novelty detection	Unknown class or distributional incompatibility	Classifier, score, or calibration rule	Class label or rejection
Open-world ReID	Target versus non-target people, possibly over time	Encoder, verifier, or discovery module	Verification, rejection, and sometimes enrollment
SDSE unknown-person detection	Identity absence relative to the current gallery	Auxiliary profile evaluator; ReID encoder frozen	Rejection score; accepted-query ranking unchanged
SDSE encoder selection	Best frozen encoder without target unknown labels	No candidate encoder update	One encoder selected before official queries

**Table 5:** Rationale for the three groups in the SDSE profile. The table gives testable motivations, not an optimality theorem.

Evidence Group	Statistics	Failure Mode Addressed
Local order	$d_{\left(1\right)}$, $d_{\left(2\right)}$, and $d_{\left(2\right)}-d_{\left(1\right)}$	A single accidental impostor match can make $d_{\left(1\right)}$ small; the second order statistic and gap expose local ambiguity.
Global distribution	Mean, standard deviation, median, maximum, and CV	A difficult known query and an unknown query may have similar nearest distances but occupy different positions relative to the complete gallery.
Candidate-conditioned consistency	SoftCV	Distances to samples of the nearest identity can be compared with distances to the remaining identities, using enrolled gallery labels without query labels.

**Table 6:** Gallery and query composition after the 50% known-identity split. Gallery and query counts are means over primary seeds 1–5; brackets give the median and interquartile range of gallery images per enrolled identity.

Dataset	Known/Unknown IDs	Gallery Images	Images/Known ID	Known Queries	Unknown Queries
Market-1501	376/375	8306±1540	16 [11, 22]	1694	1674
DukeMTMC-reID	555/555	8823±299	13 [6, 19]	1116	1112
CUHK03	350/350	2674±8	8 [8, 8]	700	700

**Table 7:** Macro open-set results over three datasets and five paired identity splits. Best values among the listed methods are bold; FPR@95 is lower-is-better.

Method	AUROC	AUPR	OSCR	FPR@95
Mahalanobis (Ledoit–Wolf)	0.610±0.008	0.607±0.012	0.284±0.008	0.892±0.016
LOF (k=20)	0.664±0.008	0.656±0.011	0.339±0.006	0.867±0.016
Energy	0.825±0.007	0.807±0.006	0.412±0.006	0.478±0.015
Mean 5-NN distance	0.857±0.004	0.841±0.005	0.423±0.007	0.469±0.012
Cosine dist. (unit norm)	0.899±0.001	0.883±0.003	0.435±0.008	0.338±0.016
Nearest distance	0.912±0.002	0.894±0.002	0.439±0.008	0.283±0.008
SDSE	0.921±0.002	0.903±0.002	0.442±0.008	0.264±0.003

**Table 8:** Per-dataset open-set results over five identity splits. Market-1501 is the representation-training reference. Bold values indicate the best mean within each dataset and metric; ties at the displayed precision are resolved using unrounded values.

Dataset	Method	AUROC	AUPR	OSCR	FPR@95
Market-1501	Energy	0.984±0.001	0.980±0.003	0.828±0.009	0.063±0.005
	Cosine dist. (unit norm)	0.992±0.001	0.986±0.003	0.835±0.009	0.027±0.004
	Nearest distance	0.993±0.001	0.991±0.002	0.835±0.009	0.025±0.001
	SDSE	0.993±0.002	0.988±0.003	0.835±0.009	0.023±0.005
DukeMTMC-reID	Energy	0.728±0.016	0.696±0.015	0.355±0.005	0.705±0.028
	Cosine dist. (unit norm)	0.837±0.003	0.805±0.008	0.413±0.010	0.473±0.018
	Nearest distance	0.856±0.004	0.823±0.006	0.420±0.011	0.430±0.020
	SDSE	0.869±0.003	0.838±0.006	0.428±0.011	0.431±0.021
CUHK03	Energy	0.764±0.008	0.745±0.010	0.052±0.006	0.666±0.016
	Cosine dist. (unit norm)	0.867±0.008	0.858±0.009	0.058±0.008	0.516±0.047
	Nearest distance	0.886±0.005	0.868±0.007	0.061±0.007	0.395±0.012
	SDSE	0.900±0.006	0.882±0.009	0.064±0.007	0.339±0.015

**Table 9:** Confirmatory paired comparison of SDSE and nearest distance over 20 identity splits. Each observation is the three-dataset macro difference for one seed. FPR@95 is oriented as nearest minus SDSE; all other differences are SDSE minus nearest.

Metric	Mean Favorable Difference	95% Bootstrap CI	Wilcoxon *p*	Positive Splits
AUROC	0.00952	[0.00867, 0.01041]	$1.91\times 10^{-6}$	20/20
AUPR	0.00937	[0.00822, 0.01060]	$1.91\times 10^{-6}$	20/20
OSCR	0.00365	[0.00341, 0.00389]	$1.91\times 10^{-6}$	20/20
FPR@95 reduction	0.02031	[0.01586, 0.02492]	$1.91\times 10^{-6}$	20/20

**Table 10:** Functional role, empirical evidence, and interpretation of the framework components.

Component	Functional Role	Evidence and Interpretation
Nine-statistic profile	Common gallery-distribution representation	Eight- and nine-statistic profiles obtain macro AUROC 0.9208 and 0.9209; local and global evidence matter, while the ninth term adds little in aggregate.
SDSE evaluator	Source-episodic gallery-presence rule	Against nearest distance over 20 splits, AUROC improves by 0.00952 and OSCR by 0.00365; both effects are positive in 20/20 paired macro splits.
UCG	Optional operating-point safeguard	The adaptive rule selects energy alone in 14/15 trials and matches the fixed energy gate in mean acceptance and rejection; Mahalanobis fusion adds no measured benefit.
Frozen-encoder selection	Gallery-only candidate ranking	Six-candidate mean regret is 0.00150 for AUROC and 0.00549 for OSCR; the result establishes within-bank feasibility, not architecture-general validity.
Integrated pipeline	Separation of learning, calibration, and inference	Frozen encoding, scoring, target-known calibration, optional gating, and pre-query selection share a gallery interface while retaining distinct evidence and failure modes.

**Table 11:** Controlled gallery and query sensitivity over three datasets and five paired seeds (15 dataset–seed trials per row). Gains are SDSE minus nearest distance. AUPR varies with the unknown-query prevalence by definition.

Factor	Level	Gallery IDs	Images/ID	SDSE AUROC	ΔAUROC	SDSE AUPR	SDSE OSCR/ΔOSCR
Gallery identities	Small (25% of base known IDs)	107	13.58	0.9491	+0.0099	0.9608	0.5358/+0.0044
	Medium (50%)	214	14.68	0.9388	+0.0120	0.9291	0.4850/+0.0042
	Large (100%)	427	15.21	0.9199	+0.0098	0.9040	0.4390/+0.0040
Images per identity	One	427	1.00	0.7078	+0.0133	0.6641	0.2669/+0.0154
	At most two	427	2.00	0.7705	+0.0170	0.7268	0.2924/+0.0153
	All available	427	15.21	0.9182	+0.0098	0.8997	0.4387/+0.0043
Identity composition	Balanced: one image/ID	427	1.00	0.6976	+0.0120	0.6507	0.2624/+0.0147
	Long tail: alternating one/all	427	7.39	0.8008	+0.0203	0.7427	0.3526/+0.0144
Unknown-query proportion	25%	427	15.21	0.9171	+0.0077	0.7752	0.4382/+0.0038
	50%	427	15.21	0.9182	+0.0098	0.8997	0.4387/+0.0043
	75%	427	15.21	0.9203	+0.0080	0.9634	0.4364/+0.0038

**Table 12:** Frozen-encoder selection over six candidates, five seeds, and equal target-gallery episode budgets. Gain is selected SDSE minus the nearest distance for the same encoder.

Dataset	Criterion	Spearman	Top-1 Hit	Mean Regret	Max Regret	SDSE Gain
Market-1501	AUROC	0.943	0.60	0.00027	0.00104	0.00014±0.00090
DukeMTMC-reID	AUROC	0.977	0.60	0.00158	0.00548	0.01305±0.00347
CUHK03	AUROC	0.966	0.40	0.00264	0.00808	0.01704±0.00446
Market-1501	OSCR	0.954	0.20	0.01095	0.01716	0.00033±0.00040
DukeMTMC-reID	OSCR	0.977	0.60	0.00192	0.00892	0.00802±0.00084
CUHK03	OSCR	0.886	0.40	0.00359	0.01119	0.00311±0.00178

**Table 13:** Known-query retrieval of the fixed OSNet representation over five splits.

Dataset	Rank-1	mAP
Market-1501	0.838±0.009	0.630±0.010
DukeMTMC-reID	0.453±0.013	0.254±0.006
CUHK03	0.066±0.008	0.072±0.004

**Table 14:** Macro decision-level ablation over five splits. Gate precision and recall describe queries flagged by the auxiliary risk gate; they are not threshold-free scoring metrics.

Decision Rule	Unknown Reject	Known Accept	Gate Precision	Gate Recall
SDSE	0.380±0.079	0.970±0.003	–	–
SDSE + Mahalanobis gate (α=0)	0.413±0.072	0.914±0.003	0.512±0.024	0.061±0.005
SDSE + fixed fusion (α=0.5)	0.451±0.062	0.936±0.004	0.788±0.013	0.150±0.003
SDSE + energy gate (α=1)	0.472±0.055	0.946±0.004	0.865±0.005	0.247±0.023
SDSE + adaptive UCG	0.472±0.055	0.946±0.004	0.865±0.005	0.242±0.021

**Table 15:** Nested and grouped profile analysis over the same three datasets and five paired splits. Datasets are first averaged within each seed; entries are mean ± sample standard deviation across seeds. Bold values indicate the best mean for each metric; ties at the displayed precision are resolved using unrounded values.

Profile	AUROC	AUPR	OSCR	FPR@95
Nearest distance (1)	0.9118±0.0016	0.8941±0.0025	0.4386±0.0078	0.2835±0.0085
Local order statistics (3)	0.9139±0.0013	0.8966±0.0022	0.4386±0.0078	0.2830±0.0107
Global distribution only (5)	0.5635±0.0040	0.5596±0.0081	0.2549±0.0069	0.9238±0.0068
Local + global (8)	0.9208±0.0016	0.9028±0.0016	0.4426±0.0078	0.2637±0.0047
Full profile (9)	0.9209±0.0017	0.9028±0.0018	0.4424±0.0078	0.2643±0.0025

## Data Availability

Market-1501, DukeMTMC-reID, and CUHK03 are publicly available from their respective benchmark providers. The SDSE implementation, random-seed protocol, evaluation scripts, and qualitative-figure generator are openly available at https://github.com/silkyrose/SDSE-OpenSet-ReID (accessed on 23 August 2026). Dataset images, trained weights, and exported embeddings are not redistributed; users should obtain the datasets from their providers and follow the corresponding terms of use.

## Abbreviations

The following abbreviations are used in this manuscript:

ReID	Person re-identification
SDSE	Source-Episodic Distance-Statistics Evaluator
AUROC	Area under the receiver operating characteristic curve
AUPR	Area under the precision–recall curve
CV	Coefficient of variation
mAP	Mean average precision
OSCR	Open-set classification rate
FPR@95	False-positive rate at 95% true-positive rate
UCG	Unknown-Candidate Gate
LOO	Leave-one-out
